# Sexual behaviour change following HIV testing services: a systematic review and meta‐analysis

**DOI:** 10.1002/jia2.25635

**Published:** 2020-11-08

**Authors:** Ruchi Tiwari, Jiayu Wang, Hannah Han, Ngozi Kalu, Lee B Sims, David A Katz, Barbara Burke, Adino T Tsegaye, Kayla A Carter, Sophie Freije, Boya Guo, Mohamed Albirair, Magdalena Barr‐DiChiara, Rachel Baggaley, Muhammad S Jamil, Kafui Senya, Cheryl Johnson, Christine M Khosropour

**Affiliations:** ^1^ Department of Epidemiology University of Washington Seattle WA USA; ^2^ Department of Global Health University of Washington Seattle WA USA; ^3^ Department of Infectious Disease Epidemiology London School of Hygiene and Tropical Medicine London United Kingdom; ^4^ School of Public Health Imperial College London London United Kingdom; ^5^ Global HIV, Hepatitis and STIs programme World Health Organization Geneva Switzerland; ^6^ Communicable Diseases Cluster World Health Organization Accra Ghana

**Keywords:** HIV testing, sexual behaviour change, condom‐protected sex, number of sexual partners, systematic review, meta‐analysis

## Abstract

**Introduction:**

Learning one’s HIV status through HIV testing services (HTS) is an essential step toward accessing treatment and linking to preventive services for those at high HIV risk. HTS may impact subsequent sexual behaviour, but the degree to which this varies by population or is true in the setting of contemporary HIV prevention activities is largely unknown. As part of the 2019 World Health Organization Consolidated Guidelines on HTS, we undertook a systematic review and meta‐analysis to determine the effect of HTS on sexual behaviour.

**Methods:**

We searched nine electronic databases for studies published between July 2010 and December 2019. We included studies that reported on at least one outcome (condom use [defined as the frequency of condom use or condom‐protected sex], number of sex partners, HIV incidence, STI incidence/prevalence). We included studies that prospectively assessed outcomes and that fit into one of three categories: (1) those evaluating more versus less‐intensive HTS, (2) those of populations receiving HTS versus not and (3) those evaluating outcomes after versus before HTS. We conducted meta‐analyses using random‐effects models.

**Results and discussion:**

Of 29 980 studies screened, 76 studies were included. Thirty‐eight studies were randomized controlled trials, 36 were cohort studies, one was quasi‐experimental and one was a serial cross‐sectional study. There was no significant difference in condom use among individuals receiving more‐intensive HTS compared to less‐intensive HTS (relative risk [RR]=1.03; 95% CI: 0.99 to 1.07). Condom use was significantly higher after receiving HTS compared to before HTS for individuals newly diagnosed with HIV (RR = 1.65; 95% CI: 1.36 to 1.99) and marginally significantly higher for individuals receiving an HIV‐negative diagnosis (RR = 1.63; 95% CI: 1.01 to 2.62). Individuals receiving more‐intensive HTS reported fewer sex partners at follow‐up than those receiving less‐intensive HTS, but the finding was not statistically significant (mean difference = −0.28; 95% CI: −3.66, 3.10).

**Conclusions:**

Our findings highlight the importance of using limited resources towards HTS strategies that focus on early HIV diagnosis, treatment and prevention services rather than resources dedicated to supplementing or enhancing HTS with additional counselling or other interventions.

## 1 INTRODUCTION

HIV testing and knowledge of one’s status is an essential first step towards linkage to HIV treatment, prevention and care [1]. Early linkage to antiretroviral therapy (ART) following an HIV‐positive diagnosis reduces HIV‐related mortality and morbidity and prevents HIV transmission from those who maintain viral suppression [2, 3, 4, 5, 6, 7, 8]. Linking HIV‐negative individuals to relevant prevention interventions (e.g. pre‐exposure prophylaxis [PrEP]) reduces the risk of HIV acquisition.

Counselling and messaging delivered through HIV testing services (HTS) may also offer an opportunity to influence subsequent sexual behaviour and thereby affect HIV acquisition and transmission [9]. These changes in behaviour may be attributable to counselling received as part of HTS, the act of testing, or knowledge of one’s HIV serostatus. An early systematic review on behaviour change following HTS, which included studies conducted between 1985 and 1997, found HTS was associated with reductions in condomless sex among HIV‐positive participants and serodiscordant couples, but not among HIV‐negative individuals [9]. More recently, evidence from low‐ and middle‐income countries has highlighted associations between HTS and reductions in the number of sex partners, increases in condom‐protected sex among HIV‐positive individuals [10] and risk reduction among HIV‐negative serodiscordant partners [11]. However, there has been little assessment of the impact of HTS on sexual behaviour in the present era of new modes of HTS (e.g. self‐testing), scale‐up of ART and the introduction of prevention options, such as PrEP – particularly among key populations (e.g. men who have sex with men [MSM]) or by partner type (e.g. primary/non‐primary) [9, 10, 11]. Thus, it remains unclear the extent to which contemporary HTS affects subsequent behaviour change in different populations.

In 2019, to update the World Health Organization (WHO) Consolidated Guidelines on HTS, WHO identified sexual behaviour change following HTS as an important area for review. This provided an opportunity to update the previous evidence presented by Fonner and colleagues [10]. The primary objective of this study was to synthesize the evidence on the effect of HTS on sexual behaviour.

## 2 METHODS

### 2.1 Guiding frameworks

This review protocol followed PRISMA guidelines [12].

### 2.2 Inclusion criteria

We included studies published in a peer‐reviewed journal or conference abstract between 1 July 2010 and 31 December 2019. The start date for inclusion represents the end date of the previous review by Fonner and colleagues [10]. We searched nine electronic databases and four conference abstract databases/books (Data S1). Studies were eligible if they prospectively compared outcomes of interest, fit into one of three *a priori* exposure/comparison categories, and reported one or more outcomes. These exposure/comparison categories and outcomes are listed in Table 1.

**Table 1:** Exposure/comparison categories and outcomes included in the review of sexual behaviour change following HTS

Exposure/comparison categories (listed as exposure versus comparator)
Studies that included more intensive HTS versus less intensive HTS (i.e. studies that compared two HTS interventions with different components included, such as HTS with additional counselling sessions versus standard‐of‐care HTS)^a^ Studies that included individuals who received any HTS versus no HTS Studies that included outcomes post‐HTS (i.e. after individuals were newly diagnosed HIV negative or HIV positive) versus pre‐HTS (i.e. when individuals were living without known HIV). This group of studies compared outcomes from the same group of individuals before and after receiving HTS^b^
Outcomes
Condom use (defined as the frequency of condom use or condom‐protected sex, e.g. always/sometimes use condoms versus never) Number of sex partners HIV incidence after HIV testing (proxy for change in behaviour after testing) STI incidence/prevalence after HIV testing (proxy for change in behaviour after testing)

HTS, HIV testing services; STI, sexually transmitted infections.

^a^ To standardize comparisons across studies, standard‐of‐care HTS was always considered to be “less” intensive, even in studies where the “more” intensive intervention (e.g. HIV self‐testing) may have been operationalized as less intensive or abbreviated compared to standard‐of‐care HTS

^b^ for studies that examined outcomes among individuals post‐HTS compared to pre‐HTS, we required that outcomes were ascertained in a manner that appropriately captured the period prior to HTS (i.e. before individuals knew their current HIV status) and after HTS (i.e. after individuals became aware of their HIV status).

### 2.3 Quality assessment

We assessed risk of bias for individual randomized controlled trials (RCT) and cluster‐RCTs using the Cochrane Collaboration’s tool [13]. We conducted a quality assessment for cohort studies and pre‐post studies using the National Institutes of Health Study Quality Assessment Tools for observational cohort studies and before‐after (pre‐post) studies with no control group respectively [14].

### 2.4 Data abstraction and analysis

A team of trained reviewers were involved in the review process. Search results from each database were merged and duplicate citations were removed. We used Covidence (Veritas Health Innovation Ltd, Melbourne, Australia) for screening and extraction. At least two reviewers independently screened titles and abstracts of all search results, reviewed full‐text articles for those abstracts receiving two votes for inclusion and extracted relevant data. A third reviewer resolved conflicts. Non‐English articles (n = 12) were reviewed for inclusion by WHO staff, but upon translation, these studies were deemed ineligible. The studies were reviewed from June 2018 to September 2020. The review was completed by September 2020.

All studies were categorized into one of three exposure/comparison categories (Table 1). To the extent possible, outcomes were stratified based on the following *a priori* strata: HIV status of the participant, population (MSM, people who inject drugs [PWID], adolescent girls and young women [AGYW], pregnant women, female sex workers [FSW]), sex partner type (primary, non‐primary), sex partner HIV status (HIV positive, HIV negative, unknown HIV status) and type of sex with sex partner (vaginal or anal). We only conducted stratum‐specific meta‐analyses when the studies were comparable. For all studies, we extracted data from the first follow‐up time point unless otherwise noted. Due to heterogeneity in the follow‐up time points (e.g. six months, twelve months) and the recall period for behavioural outcomes (e.g. past 30 days, past three months), we did not stratify results by these factors.

We conducted meta‐analyses using random‐effects models for outcomes measured comparably for two outcomes: (1) condom use, defined as the frequency of condom use or condom‐protected sex (henceforth referred to as “condom use”) and (2) number of sex partners. For condom use, estimates from included studies were converted to a common metric of relative risk (RR) using dichotomous outcome variables. For comparability across studies, we did not consider the adjusted estimates that were reported, and instead utilized raw Ns from the studies to calculate summary estimates (e.g. the number of individuals who reported condom use out of the total number of individuals who reported engaging in sexual activity). If condom use data were reported as categorical, we dichotomized categories (e.g. always vs. not always). For all cluster‐RCTs included in the meta‐analysis, we used the intra‐cluster correlation coefficient [ICC] of 0.026 obtained from a previous study to take into account the design effect using the formula: design effect = 1 + (cluster size‐1) × ICC [15]. For continuous outcomes, we calculated the mean difference for studies in which the mean and standard errors were reported.

Meta‐analysis and data summary were conducted using RevMan and R. Studies were excluded from meta‐analyses if condom use could not be converted to an RR or could not be determined (e.g. studies that reported on sex *acts*), if complete outcome data were not available (e.g. no standard error/standard deviation for number of sex partners), or if there was heterogeneity in how outcomes were reported (e.g. number of sex partners reported categorically). These studies and all other outcomes were summarized descriptively.

## 3 RESULTS

**Figure 1:**
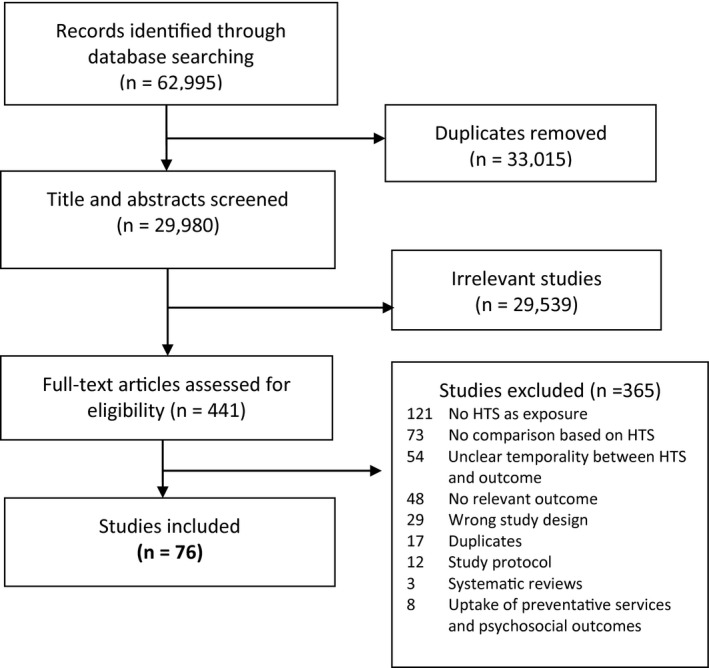
PRISMA diagram for systematic review of sexual behavior change following HIV testing services (HTS).

We screened 29 980 titles and abstracts and identified 441 full‐text articles, of which 76 were included in the review (Figure 1). A summary of these 76 studies is provided in Table 2, and outcome categories for these studies are summarized in Table 3.

**Table 2:** Summary of study characteristics for studies included in systematic review of sexual behaviour change following HTS, 2010 to 2019, by exposure/comparator category and outcome (N = 76 studies)

Description	More versus Less (N = 36)	HTS versus no HTS (N = 6)	Pre/post (N = 34)
Study characteristics	N (%)	N (%)	N (%)
Study design			
Individual RCT	22 (61)	1 (17)	0 (0)
Cluster RCT	14 (39)	1 (17)	0 (0)
Cohort	0 (0)	4 (67)	32 (94)
Serial cross‐sectional	0 (0)	0 (0)	1 (3)
Quasi‐experimental	0 (0)	0 (0)	1 (3)
WHO region			
African region	13 (36)	4 (67)	17 (50)
European region	0 (0)	1 (17)	0 (0)
Region of the Americas	14 (39)	0 (0)	9 (27)
South East Asia region	1 (3)	0 (0)	2 (6)
Western Pacific region	7 (19)	1 (17)	6 (18)
>1 WHO regions	1 (3)	0 (0)	0 (0)
World Bank income group			
High income	16 (44)	1 (17)	10 (29)
Middle income	13 (36)	2 (33)	11 (32)
Low income	4 (11)	3 (50)	11 (32)
>1 income group	3 (8)	0 (0)	2 (6)
Population included			
General population^a^	12 (33)	1 (17)	19 (56)
Female sex workers	4 (11)	1 (17)	3 (9)
Men who have sex with men	14 (39)	1 (17)	11 (32)
People who inject drugs	4 (11)	0 (0)	0 (0)
Pregnant women	2 (6)	0 (0)	1 (3)
Other key populations	0 (0)	3 (50)^b^	0 (0)

HTS, HIV testing services; RCT, randomized controlled trials; WHO, World Health Organization.

^a^ Engaged in opposite‐sex partnerships and do not fit into another category

^b^ includes adolescent girls/young women aged 13 to 22 (n = 1), male and female youth aged 15 to 24 (n = 1) and cross‐border truck drivers (n = 1).

**Table 3:** Outcomes and geographic location of studies included in systematic review and meta‐analysis of sexual behaviour change following HTS, 2010 to 2019, by exposure versus comparator category

Exposure versus Comparator	Outcome	Study	Location
More versus less intensive HTS (n = 36 studies)	Frequency of condom use/condom‐protected sex	Arnold *et al*., 2019	USA
		Coates *et al*., 2014	South Africa, Tanzania, Zimbabwe, Thailand
		Coffin *et al*., 2014	USA
		Crosby *et al*., 2019	USA
		Daniels *et al*., 2014	South Africa, Tanzania, Zimbabwe
		Doherty *et al*., 2013	South Africa
		Dong *et al*., 2019	China
		Duflo *et al*., 2019	Kenya
		El‐Bassel *et al*., 2019	USA
		Go *et al*., 2013	Vietnam
		Go *et al*., 2015	Vietnam
		Hao *et al*., 2012	China
		Hawk *et al*., 2013	USA
		Homsy *et al*., 2019	Uganda
		Jamil *et al*., 2017	Australia
		Katz *et al*., 2018	USA
		Kerrigan *et al*., 2019	Tanzania
		Kuteesa *et al*., 2019	Uganda
		Maman *et al*., 2014	South Africa
		McMahon *et al*., 2015	USA
		Metsch *et al*., 2012	USA
		Metsch *et al*., 2013	USA
		Mimiaga *et al*., 2017	India
		Mimiaga *et al*., 2019b	USA
		Ortblad *et al*., 2019	Uganda
		Passaro *et al*., 2020	Peru
		Starks *et al*., 2019	USA
		Wang *et al*., 2018	Hongkong
		Wanyenze *et al*., 2013	Uganda
		Wechsberg *et al*., 2019	South Africa
		Wray *et al*., 2019	USA
		Zhu *et al*., 2019	China
	Number of sex partners	Coffin *et al*., 2014	USA
		Dong *et al*., 2019	China
		Duflo *et al*., 2019	Kenya
		El‐Bassel *et al*., 2019	USA
		Hawk *et al*., 2013	USA
		Katz *et al*., 2018	USA
		Metsch *et al*., 2013	USA
		Mimiaga *et al*., 2019b	USA
		Oldenburg *et al*., 2018	Zambia
		Ortblad *et al*., 2019	Uganda
		Wang *et al*., 2018	Hongkong
		Wray *et al*., 2019	USA
	HIV incidence	Coates *et al*., 2014	South Africa, Tanzania, Zimbabwe, Thailand
		Dong *et al*., 2019	China
		Go *et al*., 2013	Vietnam
		Go *et al*., 2015	Vietnam
		Hao *et al*., 2012	China
		Havlir *et al*., 2019	Kenya, Uganda
		Homsy *et al*., 2019	Uganda
		Kerrigan *et al*., 2019	Tanzania
		Makhema 2019	Bostwana
		Metsch *et al*., 2013	USA
		Passaro *et al*., 2020	Peru
	STI incidence	Dong *et al*., 2019	China
		Duflo *et al*., 2019	Kenya
		Hao *et al*., 2012	China
		Homsy *et al*., 2019	Uganda
		Katz *et al*., 2018	USA
		Maman *et al*., 2014	South Africa
		Metsch *et al*., 2013	USA
		Passaro *et al*., 2020	Peru
Received HTS versus did not receive HTS (n = 6 studies)	Frequency of condom use/condom‐protected sex	Baird *et al*., 2014	Malawi
		Cawley *et al*., 2014	Tanzania
		Lau *et al*., 2010	China
	Number of sex partners	Baird *et al*., 2014	Malawi
		Cawley *et al*., 2014	Tanzania
	HIV incidence	Braunstein *et al*., 2011	Rwanda
		Cawley *et al*., 2014	Tanzania
		Furegato *et al*., 2018	United Kingdom
		Rosenberg *et al*., 2013	South Africa
Post‐HTS versus pre‐ HTS (n = 34 studies)	Frequency of condom use/condom‐protected sex	Azuogu *et al*., 2019	Nigeria
		Bao *et al*., 2014	China
		Becker *et al*., 2014	Malawi
		Bui *et al*., 2019	Vietnam
		Calvo *et al*., 2015	Peru
		Coulaud *et al*., 2019	Mali, Côte d’Ivoire, Burkina Faso and Togo
		Cremin *et al*., 2010	Zimbabwe
		Deschamps *et al*., 2016	Haiti, Dominican Republic, Puerto Rico
		Dulli *et al*., 2019	Kenya
		Fedor *et al*., 2015	Malawi
		Fiorillo *et al*., 2012	Tanzania
		Gilbert *et al*., 2018	Canada
		Gorbach *et al*., 2018	USA
		Guo *et al*., 2013	China
		Hiransuthikul *et al*., 2019	Thailand
		Huan *et al*., 2013	China
		Khosropour *et al*., 2016	USA
		Kiene *et al*., 2010	Uganda
		Koblin *et al*., 2013	USA
		Kwan *et al*., 2016	Hong Kong
		Lin *et al*., 2013	China
		Mulogo *et al*., 2013	Uganda
		Möller *et al*., 2015	Kenya
		Nuwaha *et al*., 2013	Uganda
		Pence *et al*., 2013	Tanzania
		Rosenberg *et al*., 2013	South Africa
		Rosenberg *et al*., 2017	Malawi
		Salway *et al*., 2019	Canada
		Samayoa *et al*., 2010	Guatemala
		Tang *et al*., 2016	China
		Venkatesh *et al*., 2011	South Africa, Zimbabwe
		Wall *et al*., 2016	Zambia
	Number of sex partners	Azuogu *et al*., 2019	Nigeria
		Bao *et al*., 2014	China
		Braunstein *et al*., 2011	Rwanda
		Cremin *et al*., 2010	Zimbabwe
		Deschamps *et al*., 2016	Haiti, Dominican Republic, Puerto Rico
		Gorbach *et al*., 2018	USA
		Hiransuthikul *et al*., 2019	Thailand
		Koblin *et al*., 2013	USA
		Kwan *et al*., 2016	Hong Kong
		Mulogo *et al*., 2013	Uganda
		Möller *et al*., 2015	Kenya
		Samayoa *et al*., 2010	Guatemala
		Vallabhaneni *et al*., 2013	USA
		Venkatesh *et al*., 2011	South Africa, Zimbabwe
	STI Incidence	Calvo *et al*., 2015	Peru
		Hiransuthikul *et al*., 2019	Thailand

HTS, HIV testing services; STI, sexually transmitted infection; USA, United States of America.

### 3.1 Exposure/comparator categorization 1: More‐intensive versus less‐intensive HTS

Thirty‐six studies examined more‐intensive HTS versus less‐intensive HTS. All were either individual‐RCT (n = 22) or cluster‐RCT (n = 14) (Table 2). The specific population, intervention and outcomes for these studies are summarized in Table 4.

**Table 4:** Summary of studies (location, population, design, exposure/comparator and outcomes) included in systematic review of sexual behaviour change following HTS, 2010 to 2019, by exposure versus comparator category

Study	Location		Population^a^	Study design	Description of exposure (E) and comparator (C)	Outcomes reported
More versus less intensive HIV Testing Services (HTS)						
Arnold *et al*., 2019 [16]	USA		HIV negative, HIV positive and HIV status unknown African American cis‐male aged >18 years, reported sex with at least one male and one female in past 12 months (N = 396)	RCT: individual	E: HTS + four risk reduction counselling sessions, all tailored to African American men who have sex with both men and women C: Standard of care HTS tailored to African American men who have sex with both men and women	▪ Number of condomless sex acts with any partner, primary partner, casual partner, male partner, primary male partner, casual male partner, female partner, primary female partner and casual female partner (all mean) in the past three months
Coates *et al*., 2014 [17]	South Africa, Tanzania, Zimbabwe, Thailand		Communities randomized; participants were HIV‐negative and HIV‐positive men and women aged 18 to 32 (N = 53,997)	RCT: cluster	E: Community‐based HTS (included community mobilization, easy testing access, post‐test support services, real‐time performance feedback) C: Standard HTS	▪ Monthly number of condomless sex acts (mean) ▪ Incident HIV (% new infections)
Coffin *et al*., 2014 [18]	USA		HIV‐negative MSM aged >18 years who reported condomless anal sex under the influence of harmful substance in past three months (N = 307)	RCT: individual	E: HIV testing + personalized cognitive counselling C: HIV testing	▪ Number of condomless anal sex acts, past three months (mean) ▪ Number of condomless anal sex partners, past three months (mean)
Crosby *et al*., 2019 [19]	USA		HIV‐positive and HIV‐negative African American male aged 15 to 29 years; reported anal sex with a male partner at least once in past six months (N = 277)	RCT: individual	E: HIV testing + male sexual health programme tailored to promote condom use + STI assessment C: HIV testing + STI assessment	▪ Condomless insertive anal sex and receptive anal sex among baseline HIV‐negative participants (%) in the past three months
Daniels *et al*., 2014^b^ [20]	South Africa, Tanzania, Zimbabwe		Communities randomized; participants were HIV‐positive and HIV‐negative men aged 18 to 32 who experienced childhood sexual or physical abuse (N = 904)	RCT: cluster	E: Community‐based HTS (included community mobilization, easy testing access, post‐test support services, real‐time performance feedback) C: Standard HTS	▪ Always used condoms for sex, past six months (%)
Doherty *et al*., 2014 [21]	South Africa		Communities randomized; participants were HIV‐negative and HIV‐positive men and women aged >14 years (N = 4,154)	RCT: cluster	E: Counsellor‐delivered home‐based HTS C: HTS at local clinics	▪ Condom use at last sex (%)
Dong *et al*., 2019 [22]	China		Pair matched randomization of 12 cities in 3 provinces; HIV‐negative FSWs aged >18 years who charged low fees (appx 12 USD per vaginal sex act) (N = 1024 FSWs)	RCT: cluster	E: Community‐based comprehensive intervention (including intensive HIV and syphilis testing, condom promotion, reimbursement for syphilis treatment costs and free ART) for 24 months C: Standard of care (annual HIV/syphilis testing + condom distribution and referral for HIV/STI infection)	▪ Condom‐protected sex with clients (%) at follow‐up ▪ Number of sexual partners (%, more than 5) per day ▪ Incident HIV (%) ▪ Incident syphilis (%)
Duflo *et al*., 2019 [23]	Kenya		HIV‐positive and HIV‐negative young people aged 17 to 24 years; attended at least grade 6 (N = 10245)	RCT: individual	E: HTS nearby or at participant’s homes by trained providers + free male condoms (50 packages containing 3 condoms each) C1: HTS nearby or at participant’s homes by trained providers C2: Free male condoms (50 packages containing 3 condoms each) C3: No intervention (Access to HTS at local clinics)	▪ Incident HSV‐2 among male and among female (incident rate) ▪ Incident HSV‐2 among male and among female (%) ▪ Condomless sex among males and among females (%), last sex ▪ Number of partners among male and among female (mean) in past six months
El‐Bassel *et al*., 2019 [24]	USA		Heterosexual couples were randomized; HIV‐positive and HIV‐negative men and women; Male partners involved in drug and mandated to community supervision, both partners>= 18 years and at least one partner reported having condomless sex with the other in the past 90 days (N = 230 couples)	RCT: cluster	E: Couple based HIV testing and counselling + five weekly risk reduction sessions C: Individual rapid oral HIV or STI testing, counselling and referral (one session)	▪ Condomless vaginal and/or anal sex events with primary female partner (mean) in the past 90 days ▪ Number of sexual partners (mean) in the past 90 days
Go *et al*., 2013 [25]	Vietnam		HIV‐negative male PWID and their network members aged >18 years; network members injected drugs with or had sexual intercourse with the index in the past three months (N = 419)	RCT: individual	E: HTS + six small group peer educator‐training sessions + three booster sessions C: HTS	▪ Condomless sex, past three months (%) ▪ Incident HIV (% new infections)
Go *et al*., 2015 [26]	Vietnam		HIV‐infected male PWID and their HIV negative injecting network members aged >18 years old and interacted at least once a week (N = 184)	RCT: individual	E: Individual‐level post‐test counselling and skill‐building support groups C: Standard of care HTS	▪ Incident HIV (% new infections and incidence rate) ▪ Condomless sex, past three months (%)
Hao *et al*., 2012 [91]	China		HIV‐negative MSM aged >18 years old (N = 295)	RCT: individual	E: HIV testing + video narrated by HIV‐positive Chinese MSM + enhanced post‐test counselling + bracelet as a reminder for safer sex C: Standard of care HTS	▪ Incident HIV (incidence rate) ▪ Incident syphilis (incidence rate) ▪ Condomless anal sex with all partners, regular partners and casual partners, past six months (%)
Havlir *et al*., 2019 [28]	Kenya, Uganda		HIV‐positive and HIV‐negative men and women aged >15 years (N = 1,50,395)	RCT: cluster	E: Baseline HIV and multidisease testing + annual testing, eligibility for universal antiretroviral therapy and patient‐centred care C: Baseline HIV testing and multidisease testing at health fairs and national guideline–national guideline restricted antiretroviral therapy	▪ Cumulative HIV incidence at three years (Relative risk)
Hawk *et al*., 2013 [29]	USA		Party hosts randomized; participants were HIV‐negative African‐American women aged 18 to 65 (N = 149)	RCT: cluster	E: Party with HIV risk‐reduction information, assessments and referrals for addiction and domestic violence, empowerment around sexual decision‐making; HIV testing C: Party with HIV testing and information about study	▪ Number of condom‐protected vaginal sex acts, past three months (mean) ▪ Number of condom‐protected anal sex acts, past three months (mean) ▪ Number of male sex partners, past three months (mean)
Homsy *et al*., 2019 [30]	Uganda		HIV‐negative pregnant women aged 18 to 49 years (N = 820)	RCT: individual	E: Enhanced individuals or couples HIV prevention counselling every three months for up to 24 months C: Standard counselling at time of HIV re‐testing	▪ Consistent or intermittent condom use, past three months (%) ▪ Incident HIV ▪ Incident STI (CT, GC, TV, syphilis)
Jamil *et al*., 2017 [31]	Australia		HIV‐negative MSM aged >18 years old; reporting condomless anal sex or >5 male sex partners in past three months (N = 343)	RCT: individual	E: Offered 4 HIV self‐test kits at enrolment; could request free additional kits during subsequent 12 months (maximum 12 kits/year) C: Standard of care HTS	▪ Condomless anal sex with casual partners, past 12 months (%)
Katz *et al*., 2018 [32]	USA		HIV‐negative MSM aged >18 years; at high risk for HIV (N = 197)	RCT: individual	E: Received 1 HIV self‐test kit at enrolment; could request free kits (max 1 per month) during study period C: Standard of care HTS	▪ Condomless anal sex with non‐concordant partners, past three months (%) ▪ Number of condomless anal sex partners, past three months (mean) ▪ STI prevalence (% diagnosed with early syphilis or rectal, pharyngeal, or urethral GC or CT) at 12 months
Kerrigan *et al*., 2019 [33]	Tanzania		HIV‐negative and HIV‐positive FSWs aged >18 years (N = 387)	RCT: cluster	E: Venue ‐based HIV testing + community empowerment‐based model of combination HIV prevention C: Standard of care HIV services	▪ Incident HIV (%) ▪ Inconsistent condom use with clients (%) in the past 18 months
Kuteesa *et al*., 2019 [34]	Uganda		HIV‐negative and HIV‐positive men and women aged >18 years residents of a fishing community (N = 860)	RCT: cluster	E: Community‐hub based HIV testing + combination‐prevention‐package (behaviour change communication, condom promotion, VMMC and referral for ART if HIV positive) C: Standard of care HIV services	▪ Condom use throughout the study period of 15 months (%)
Makhema 2019 [35]	Bostwana		HIV‐positive and HIV‐negative men and women aged >16 years (N = 12,610)	RCT: cluster	E: Communitywide, standardized, home‐based and mobile HTS + linkage to care + ART initiation at a higher CD4 count than in standard care + increased access to VMMC C: Standard of care	▪ Incident HIV (%)
Maman *et al*., 2014 [36]	South Africa		HIV‐negative and HIV‐positive pregnant women aged >18 years; attending first antenatal visit (N = 1,480)	RCT: individual	E: HIV testing + video + enhanced pre‐ and post‐test counselling + access to legal support and support groups C: Standard of care HTS during pregnancy + two post‐partum sessions on infant health	▪ Inconsistent condom use, past 30 days (%) ▪ Incident STI (CT, GC, TV) (% new infections)
McMahon *et al*., 2015 [37]	USA		Substance using HIV‐negative women aged >18 years; had condomless sex with a primary partner in the past 30 days; enrolled with primary heterosexual partner (N = 324)	RCT: individual	E: Couple‐based HTS C: Standard of care manualized HIV counselling and testing protocol for substance users (women‐only)	▪ Percent of condom‐protected vaginal sex acts with primary partner, past three months (%) ▪ Percent of condom‐protected anal sex acts with primary partner, past three months (%) ▪ Condom use with non‐primary partner, past three months (%)
Metsch *et al*., 2012 [38]	USA		HIV‐negative men and women aged >18 years; seeking or receiving drug treatment services; no past‐year HIV testing (N = 1,281)	RCT: individual	E: On site rapid HIV testing + HIV risk‐reduction counselling C1: On site rapid HIV testing + verbal information about testing only C2: Referral for off‐site HIV testing	▪ Condomless vaginal or anal sex with primary or non‐primary partners, past six months (%)
Metsch *et al*., 2013 [39]	USA		HIV‐negative and HIV‐positive MSW, MSM, and women aged >18 years attending STD clinics (N = 5,012)	RCT: individual	E: Rapid HIV testing with individual risk‐reduction counselling (RESPECT‐2) C: Rapid HIV testing with information only	▪ Number of condomless vaginal or anal sex acts, past six months (mean) ▪ Number of sex partners and condomless sex partners, past six months (mean) ▪ Incident HIV, STI, GC, CT, syphilis (% new infections)
Mimiaga *et al*., 2017 [40]	India		HIV‐negative and HIV‐positive MSM aged >18 years; engaged in exchange sex with another man in past three months (N = 100)	RCT: individual	E: HTS + Integrated in‐person and mobile phone‐delivered counselling + daily text messaging C: Standard of care HTS	▪ Number of condomless anal sex acts with male clients and non‐paying male partners, past month (mean)
Mimiaga *et al*., 2019a [41]	USA		HIV‐negative MSM who reported having condomless anal sex within the context of crystal methamphetamine use in the past three months and who met DSM‐IV criteria for crystal methamphetamine dependence (N = 46)	RCT: individual	E: HIV testing + cognitive behaviour therapy for substance abuse + behavioural activation and sexual risk reduction counselling (13 sessions) C: HIV testing + sexual risk reduction counselling only (2 sessions)	▪ Number of condomless anal sex acts with HIV serodiscordant partner or partner whose status was unknown in the past three months (mean) ▪ Number of condomless anal sex acts with HIV serodiscordant partner or partner whose status was unknown while using meth in the past three months (mean)
Mimiaga *et al*., 2019b [42]	USA		HIV‐negative men aged 18 to 50 years, reporting anal sex with another man in the past 12 months an d condomless anal sex with another man at a private sex event in the past three months (N = 14)	RCT: individual	E: HTS + four group sessions focusing on HIV risk reduction education and skills building C: HTS	▪ Unprotected anal sex with HIV serodiscordant sex partner in the past three months (%) ▪ Number of unprotected anal sex acts in the past three months (mean) ▪ Number of male sex partners in the past three months (mean)
Oldenburg *et al*., 2018 [43]	Zambia		Peer educators were randomized; participants were HIV‐negative and HIV‐positive women aged >18 years who reported exchange sex in past month (N = 645)	RCT: cluster	E: Counselling + referral to facility HIV testing + distribution of two HIV self‐test kit from peer educator to participant C: Counselling + referral to facility HIV testing	▪ Number of non‐client sex partners, past 30 days (mean)
Ortblad *et al*., 2019 [44]	Uganda		Peer educators were randomized; participants were HIV‐negative women aged >18 years who reported exchange sex in past month (N = 960)	RCT: cluster	E: Direct provision of one HIV self‐test kit + information on HIV prevention + referral for facility HTS C1: Provision of facility coupon for collection of HIV self‐test kit + information on HIV prevention + referral for facility HTS C2: Referral for facility HTS	▪ Number of clients, past month (mean per night) ▪ Inconsistent condom use with clients, past month ▪ Number of non‐clients, past month (mean per night) ▪ Inconsistent condom use with non‐clients, past month
Passaro *et al*., 2020 [45]	Peru		HIV uninfected MSM who tested positive for rectal GC/CT (N = 101)	RCT: individual	E: HIV testing + Personalized cognitive counselling (PCC) designed to modify HIV‐related risk behaviour C: HIV testing + traditional counselling	▪ Condomless anal sex acts (mean) in the past month ▪ Incident HIV (%) ▪ Incident GC/CT (%)
Starks *et al*., 2019 [46]	USA		Couples were randomized; Either member HIV negative or unknown status and used drugs in the past 30 days and aged <30 years; Both partners aged >18 years and indicated male sex and gender (N = 70 couples)	RCT: cluster	E: Couples HIV testing and counselling (CHTC) + communication training (CT) videos + substance use module (SUM) – to reduce drug use and sexual HIV transmission risk C1: CHTC + SUM C2: CHTC + SUM + CT C3: CHTC	▪ Condomless anal sex with casual partners (Odds, 95% CI) at one month
Wang *et al*., 2018 [47]	Hongkong		HIV‐negative male aged >18 years, reported anal intercourse with a man in the last six months (N = 430)	RCT: individual	E: Mailing of self‐test kits + video promoting HIV testing, self‐testing and online real‐time instructions and counselling C: Video promoting HIV testing coupled with a list of places to get tested	▪ Condomless anal intercourse with men among those who tested for HIV (%), in the past three months ▪ Multiple male sex partners among those who tested for HIV (%) in the past three months
Wanyenze *et al*., 2013 [48]	Uganda		HIV‐negative and HIV‐positive men and women aged >18 years; patients in inpatient wards or outpatient clinics (N = 2,066)	RCT: individual	E: Abbreviated HTS^c^ C: Traditional HTS	▪ Condomless sex with potentially HIV discordant partner, past three months (%)
Wechsberg *et al*., 2019 [49]	South Africa		HIV‐positive and HIV‐negative Black African women aged ≥15 years with evidence of tacit emancipation (for aged 15 to 17 years), used substance (including alcohol) weekly for past three months, had condomless sex with a male partner in past six months (N = 641)	RCT: cluster	E: HTS + two evidence‐based gender‐focused HIV prevention intervention sessions (including education about risks of alcohol and drug use and relation to sexual risk) C: Standard of Care HTS	▪ Condom use with primary partner, last sex (adjusted OR) ▪ Condom use with casual partner or client, last sex (adjusted OR) ▪ Number of condom protected sex acts with primary partner, past month (regression coefficient) ▪ Number of condom protected sex acts with casual partner or client, past month (regression coefficient)
Wray *et al*., 2019 [50]	USA		HIV‐negative MSM aged >18 years, heavy drinkers, who sought rapid HIV testing; reported condomless anal sex with male partner of unknown HIV status in past three months (N = 40)	RCT: individual	E: Standard of care HTS + web‐based intervention that provided individualized feedback on HIV risk behaviour and alcohol use C: Standard of care HTS	▪ Number of new anal sex partners (IRR) in past three months ▪ Condomless anal sex events (IRR) in past three months ▪ High risk condomless anal sex events (IRR) in past three months
Zhu *et al*., 2019 [51]	China		HIV‐negative MSM aged >18 years, reported had unprotected anal sex with another man in the past six months and agreed to administer oral HIVST kit at baseline (N = 100)	RCT: individual	E: Distribution of two oral HIVST kits + access to a private WeChat group which provided app‐based messages and referrals to health services related to HIV C: Distribution of two oral HIVST kits only	▪ Consistent condom use with primary partner and casual or commercial partner, past six months (%, aRR) ▪ Consistent condom use during receptive anal sex and insertive anal sex, past six months (%, aRR)
Receiving HIV Testing Services versus Not Receiving HIV Testing Services						
Baird *et al*., 2014 [52]		Malawi	Adolescent girls/young women aged 13 to 22; never married; HIV negative and HIV positive (N = 1,681)	RCT: cluster	E: Home‐based HIV testing and counselling in 2009 C: Delayed home‐based HIV testing and counselling (offered in 2010)	▪ Any condomless sex, past 12 months (%) ▪ Number of sex partners, past 12 months (mean)
Braunstein *et al*., 2011 [53]		Rwanda	Non‐pregnant FSW aged >18 years; HIV negative (N = 397)	Cohort	E: Ever tested for HIV (once or >2 times) C: Never tested for HIV	▪ HIV incidence (incidence rates and HR)
Cawley *et al*., 2014 [54]		Tanzania	Men and women aged >15 years; HIV negative and HIV positive (N = 3613 and N = 2998)	Cohort; Four sero surveys	E: Use of HTS services C: No HTS services	▪ Number of sex partners in past year (% who decreased number of annual partners after last survey) ▪ Started using condoms with spouse since last survey (%) ▪ Started using condoms with regular co‐habitating partners since last survey (%) ▪ Started using condoms with casual partners since last survey (%) ▪ HIV incidence (incidence and rate ratio)
Furegato *et al*., 2018 [55]		United Kingdom	MSM aged >15 years; HIV negative (N = 37,702)	Cohort	E: HIV tested in past year (one, two, three, or four tests) C: No HIV test in past year	▪ HIV incidence in the 12 months following the HIV testing pattern observed as exposure (HR)
Lau *et al*., 2010 [27]		China	Male Hong Kong Chinese cross‐border truck drivers aged >18; reported sex with FSW or non‐regular partner (N = 301)	RCT: individual	E: HIV testing and counselling (standard HTS service) C: Educational pamphlets only (no HIV testing)	▪ Consistent condom use with FSW, past month (%) ▪ Consistent condom use with primary partners, past month (%) ▪ Consistent condom use with non‐primary partners, past month (%)
Rosenberg *et al*., 2013 [56]		South Africa	Male and female youth aged 15 to 24 years (N = 3,959)	Cohort	E: Standard HTS^d^ C: Never exposed to HTS	▪ HIV incidence (incidence rates and HR)
After (Post) versus Before (Pre) Receiving HIV Testing Services^e^						
Azuogu *et al*., 2019^f^ [57]		Nigeria	HIV‐negative and HIV‐positive residents of cantonments (N = 350)	Cohort	On site and house‐to‐house peer education and HIV testing, and HIV awareness activities rolled out community‐wide	▪ Always used condom during casual sex (%) in past three months ▪ Number of casual sexual partners (none, only one, >1) (%) in past three months
Bao *et al*., 2014 [58]		China	Men and women; newly diagnosed HIV positive (N = 608)^g^	Cohort	Standard of care HTS	▪ Condomless anal or vaginal sex with HIV negative or unknown status partners, past six months (%) ▪ Number of HIV negative or unknown‐status sex partners with whom participant had condomless anal or vaginal sex (mean)
Becker *et al*., 2014 [59]		Malawi	Man‐woman pair married or in union; women aged 15 to 49 years and men aged >15 years (N = 71); newly diagnosed HIV negative or HIV positive	Cohort	Couple HTS^h^	▪ Condom use at last sex (%), at one week follow‐up
Braunstein *et al*., 2011 [60]		Rwanda	Female sex workers aged >18 years; newly diagnosed HIV positive (N = 141)	Cohort	Standard of care HTS	▪ Number of clients per week (median)
Bui *et al*., 2019 [61]		Vietnam	HIV‐negative partners of serodiscordant couples aged >18 years (N = 134)	Cohort	Couple HTS, including immediate ART to partner	▪ Consistent condom use with the study partner (%) in past three months
Calvo *et al*., 2015 [62]		Peru	MSM and transgender women; newly diagnosed HIV positive (N = 32)	Cohort	Standard of care HTS	▪ Condomless receptive anal sex, past three months (%) ▪ Condomless insertive anal sex, past three months ▪ Number of sex partners, past three months (median and IQR) ▪ Diagnosed with rectal GC/CT (%)
Coulaud *et al*., 2019 [63]		Mali, Côte d’Ivoire, Burkina Faso and Togo	MSM aged >18 years; newly diagnosed HIV negative (N = 621)	Cohort	Comprehensive preventive package including free quarterly HTS, screening and treatment for other STIs, access to post‐exposure prophylaxis, individualized peer‐led support, condoms and lubricants	▪ Inconsistent condom use during receptive anal sex with sexual male partner of unknown HIV serostatus (%) in past six months
Cremin *et al*., 2010 [64]		Zimbabwe	Men and women aged 15 to 54 years; newly diagnosed HIV negative or HIV positive (N = 17,874)	Open cohort	Free standard of care HTS clinic; mobile clinic	▪ Consistent condom use in past two weeks with primary and non‐primary partners (coefficient) ▪ Number of new sex partners in past year (coefficient)
Deschamps *et al*., 2016 [65]		Haiti, Dominican Republic, Puerto Rico	Female sex workers aged 18 to 45 years old; newly diagnosed HIV negative (N = 799)	Cohort	Standard of care HTS	▪ Condomless vaginal sex, past six months (%) ▪ Condomless anal sex, past six months (%) ▪ Number of sex partners, past six months (median)
Dulli *et al*., 2019^f^ [66]		Kenya	HIV‐positive and HIV‐negative FSWs who received money or goods in exchange for sex in the past six months; aged 16 to 49 years; attending drop‐in centres (N = 719)	Two‐group, pre‐/post‐test quasi experiment	E: Enhanced standard health services designed to improve consistent contraceptive use and dual method use E: Standard HTS	▪ Condom use with paying partner (%) at last sex ▪ Condom use with non‐paying partner (%) at last sex
Fedor *et al*., 2015 [67]		Malawi	Men and women aged >18 years; newly diagnosed HIV negative (men: N = 595; women = 758) or HIV positive (N = 74)	Cohort	Standard HTS	▪ Increase in condom use with spouse after learning HIV status (%)
Fiorillo *et al*., 2012 [68]		Tanzania	Men and women aged >18 years; newly diagnosed HIV negative^i^ (N = 366)	Cohort	Standard of care HTS	▪ Used condoms in the past month (%); measured at time of second HIV test
Gilbert 2018 [69]		Canada	MSM aged >19 years; newly diagnosed HIV positive (N = 25)	Cohort	Standard of care HTS	▪ Condomless anal sex with serodiscordant or unknown‐status partner, past three months (%)
Gorbach *et al*., 2018 [70]		USA	MSM aged >18 years; newly diagnosed HIV positive (N = 125) or HIV negative (N = 113)	Cohort	Standard of care HTS	▪ Condomless anal sex with serodiscordant or unknown‐status partner, past three months (%)Number of sex partners, past 12 months (median, IQR) ▪ Condomless insertive anal sex, last partner (%) ▪ Condomless receptive anal sex, last partner (%)
Guo *et al*., 2013 [71]		China	MSM aged >18 years; newly diagnosed HIV positive (N = 13) or HIV negative (N = 187)	Cohort	Standard of care clinic HTS	▪ Consistent condom use, past three months (%)
Hiransuthikul *et al*., 2019 [72]		Thailand	MSM or TGW aged >18 years, reported unprotected anal sex with men at least one time or had at least 3 male sexual partners in the last six months, newly diagnosed HIV positive (N = 43) or HIV negative (N = 466)	Cohort	HTS and immediate ART	▪ Multiple sexual partners (%) in the past month ▪ Unprotected anal intercourse (%) in the past month
Huan *et al*., 2013 [73]		China	MSM aged >18 years; newly diagnosed HIV negative (N = 283 at six months)	Cohort	Standard of care HTS	▪ Condomless anal sex, past six months (%) ▪ Condomless vaginal sex, past six months (%) ▪ Condomless sex with primary partners, past six months (%) ▪ Condomless sex with casual partners, past six months (%)
Khosropour *et al*., 2016 [74]		USA	MSM STD clinic patients; newly diagnosed HIV positive (N = 43) or HIV negative (N = 281)^j^	Retrospective cohort	Standard of care HTS	▪ Condomless anal sex with HIV‐positive partners, past 12 months (%) ▪ Condomless anal sex with HIV‐negative partners, past 12 months (%) ▪ Condomless anal sex with HIV‐unknown status partners, past 12 months (%)
Kiene *et al*., 2010 [75]		Uganda	Men and women aged >18 years; newly diagnosed HIV positive (N = 28) or negative (N = 187)	Cohort	Provider‐initiated routine HTS	▪ Condomless sex with serodiscordant or unknown‐status partner, past three months (%)
Koblin *et al*., 2013 [76]		USA	Women aged 18 to 45 at high risk for HIV; newly diagnosed HIV negative (N = 799)	Cohort	Standard of care HTS	▪ Condomless vaginal sex, past six months (%) ▪ Condomless anal sex, past six months (%) ▪ Number of male sex partners, past six months (median)
Kwan *et al*., 2016 [77]		Hong Kong	MSM aged >18 years; newly diagnosed HIV positive (N = 345)	Cohort	Standard of care HTS	▪ >1 primary sex partner, past 12 months (%) ▪ >2 casual sex partners per month, past 12 months (%) ▪ Inconsistent condom use with primary partner, past 12 months (%) ▪ Inconsistent condom use with casual sex partners, past 12 months (%)
Lin *et al*., 2013 [78]		China	Men and women aged >18 years; newly diagnosed HIV positive (N = 262)	Cohort	Standard HTS	▪ Consistent condom use, at 12 months after baseline^k^; % reported as number of partners with whom participant used condoms out of total number of partners
Möller *et al*., 2015 [79]		Kenya	MSM aged 18 to 49 years who reported anal sex during follow‐up; newly diagnosed HIV negative (N = 469)	Cohort	HIV testing and regular risk reduction counselling every one or three months	▪ Number of regular and casual sex partners in past week, at 12 month follow‐up (coefficient) ▪ Condomless sex, past week, at 12‐month follow‐up (adjusted OR) ▪ Condomless anal sex in the past three months, at 12‐month follow‐up (adjusted OR)
Mulogo *et al*., 2013 [80]		Uganda	Men and women aged 18 to 59 years old; newly diagnosed HIV negative or HIV positive (N = 975)	Cohort	Facility or home‐based HTS	▪ Used condoms every time had sex, past two months (%)^l^ ▪ Reduction in the number of sex partners (%), past two months^l^
Nuwaha *et al*., 2013 [81]		Uganda	Randomly‐selected men and women aged 18 to 49 from randomly‐selected households; HIV‐negative and HIV positive^m^	Serial cross‐sectional surveys	Home‐based HTS rolled out district‐wide	▪ Condom use at last sex (%)
Pence *et al*., 2013 [82]		Tanzania	Men and women aged 18 to 65; newly diagnosed HIV positive (N = 282)	Cohort	Standard of care HTS	▪ Condomless sex in past six months (%)
Rosenberg *et al*., 2013 [83]		South Africa	Men and women; enrolled as serodiscordant couples; newly diagnosed HIV positive (N = 254)	Retrospective cohort	Standard of care HTS	▪ Reported zero condomless sex acts in past month with HIV‐negative study partner (%)
Rosenberg *et al*., 2017 [84]		Malawi	Heterosexual couples with an HIV‐positive pregnant woman (N = 90) and HIV‐negative pregnant woman (N = 47); women aged >18 years; testing newly HIV positive or negative^n^	Cohort	Couple HTS	▪ Consistent condom use with study partner, past month (%)
Salway *et al*., 2019^f^ [85]		Canada	Men and women; newly diagnosed HIV negative (N = 271)	Cohort	E: Internet‐based HIV testing	▪ Condomless anal/vaginal sex (%) in past three months ▪ Increase in condom use at post‐test compared to pre‐test (%) ▪ Change in condom use associated with treatment (aRR)
Samayoa *et al*., 2010 [86]		Guatemala	Men and women presenting for HIV testing; newly diagnosed HIV negative (N = 49) or HIV positive (N = 41)	Cohort	Standard of care HTS	▪ Number of sex partners, past three months (mean) ▪ Never engaged in condomless sex, past three months (%)
Tang *et al*., 2016 [87]		China	Heterosexual serodiscordant couples (N = 120); HIV‐negative partner tested newly HIV positive or remained HIV negative	Open cohort	Standard of care HTS	▪ Consistent condom use between couples (%)^o^
Vallabhaneni *et al*., 2013 [88]		USA	MSM aged >18 years; newly diagnosed HIV positive (N = 54)	Cohort	Standard of care HTS	▪ Number of sex partners in past three months, at six‐month follow‐up (estimated mean)
Venkatesh *et al*., 2011 [89]		South Africa, Zimbabwe	Women aged 18 to 49 years; newly diagnosed HIV positive (N = 327)^p^	Cohort (nested within RCT)	Standard of care HTS	▪ Consistent condom use (women who reported always using a condom in past three months and at last sex act) (%) ▪ >1 sex partner, past three months (%)
Wall *et al*., 2013 [90]		Zambia	Heterosexual HIV serodiscordant couples aged >18 years; male HIV positive and female HIV negative (N = 1393); male HIV negative and female HIV positive (N = 1656)	Open cohort	Couple voluntary HTS	Number of condomless sex acts in past three months (mean)

CT, *Chlamydia trachomatis;* DSM‐IV, Diagnostic and Statistical Manual of Mental Disorders; FSW, female sex workers; GC, *Neisseria gonorrhoeae;* HR, hazard ratio; HTS, HIV testing services; IQR, interquartile range; MSM, men who have sex with men; MSW, men who have sex with women; OR, odds ratio; PWID, people who inject drugs; RCT, randomized controlled trial; RR, relative risk; TGW, Transgender Women; TV, *Trichomonas vaginalis;* USA, United States of America.

^a^ The terms “newly diagnosed HIV positive” and “newly diagnosed HIV negative” refer to populations for whom the HIV result was from the HTS event. Otherwise we have described populations as “HIV negative” or “HIV positive”

^b^ subgroup analysis of RCT described in Coates *et al*., 2014

^c^ for data synthesis and meta‐analyses, we assigned the “abbreviated HTS” as the comparator and the “traditional HTS” as the exposure to match the other studies comparisons of more versus less intensive HTS

^d^ time varying exposure (i.e. if participant was HTS‐unexposed at origin but later received HTS, their person‐time was assigned accordingly)‐38% of those initially unexposed became exposed

^e^ all studies included an “exposure” of the time period prior to receiving HTS (i.e. pre‐HTS) and a “comparator” of the time period after receiving HTS (i.e. post‐HTS)

^f^ used within group difference in outcome even though a comparison group was present and between group difference was reported in these studies (Azuogu 2019, Dulli 2019 and Salway 2019)

^g^ used the complete case analysis for this study (i.e. the sub‐analysis of participants who had data at baseline and follow‐up, N = 608)

^h^ study also included couples who received couples family planning along or in conjunction with couples HTS but only those who received couple HTS alone are included in this review

^i^ study included data on one‐time testers and repeat testers but only data for repeat testers was used to examine changes in behaviour between the first and second tests;

^j^ pre‐HTS testing data reported from participants at a time when they already knew they were HIV positive

^k^ included outcomes for 12 months before diagnosis and 12 months after baseline survey and for 63% of participants, baseline survey was within one year of diagnosis

^l^ participants were asked a series of questions related to what they had done to reduce their risk of HIV/STIs

^m^ at follow‐up, only 62% reported ever testing for HIV

^n^ for most HIV‐infected women (84/90, 93%) and HIV‐infected men (56/69, 81%), the HIV‐positive diagnosis was new

^o^ time frame unclear, but likely asked about pre‐ and post‐HTS behaviours at the same timep

^p^ unit of analysis was a study visit where N = 1689 visits.

#### 3.1.1 Condom use

Of the 32 studies [16, 17, 18, 19, 20, 21, 22, 23, 24, 25, 26, 29, 30, 31, 32, 33, 34, 36, 37, 38, 39, 40, 42, 44, 45, 46, 47, 48, 49, 50, 51, 91] that reported on condom use, 21 were individual‐RCT and 11 were cluster‐RCT, though two studies reported results from the same cluster‐RCT [17, 20]. The interventions included in these studies varied (Table 4). Seventeen studies examined enhanced counselling or education sessions compared to standard HTS [16, 38, 39, 40, 42, 45, 49, 50, 91], two studies examined the distribution of HIV self‐testing kits relative to standard HTS [31, 32], two studies from the same cluster‐RCT examined community‐based HTS [17, 20] along with two other studies [22, 34], one study included couple HTS relative to standard HTS [37], one study examined counsellor‐delivered home‐based HTS versus clinic‐based HTS [21] and one study examined abbreviated HTS compared to standard HTS [48].

**Figure 2:**
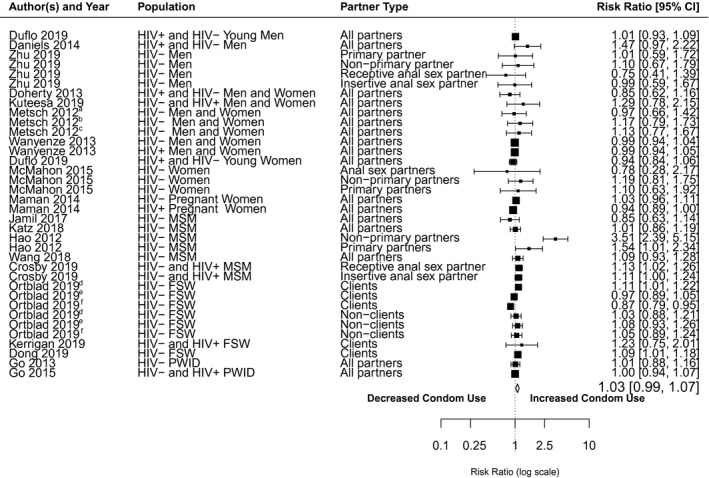
Forest plot of frequency of condom use/condom‐protected sex for studies of more intensive HIV testing services (HTS) versus less intensive HTS. The black squares represent study estimates, the lines 95% CI. The size of the squares represents a study’s weight in the meta‐analysis. The summary effect estimate is displayed as the diamond symbol. The effect estimates obtained from six cluster randomized trials, Daniels 2014, Doherty 2013, Ortblad 2019, Dong 2019, Kerrigan 2019, Kuteesa 2019, were adjusted for design effect. Random‐effect models was used to aggregate effect sizes. CI, confidence interval; FSW, female sex workers; MSM, men who have sex with men; PWID, people who inject drugs. ^a^HTS + risk reduction counseling versus HTS. ^b^HTS versus referral for off‐site HTS. ^c^HTS + risk reduction counseling versus referral for off‐site HTS. ^d^Direct provision of one HIV self‐test kit versus provision of facility coupon for collection of HIV self‐test kit. ^e^Direct provision of one HIV self‐test kit versus referral for facility HTS. ^f^Provision of facility coupon for collection of HIV self‐test kit versus referral for facility HTS

Nineteen studies were included in the meta‐analysis (Figure 2). After disaggregation by participant HIV status, sex and partner type, there were a total of 36 estimates from 19 studies. There was no significant difference in condom use after receipt of HTS among individuals receiving more‐intensive HTS compared to those receiving less‐intensive HTS (RR = 1.03; 95% Confidence Interval [CI]=0.99 to 1.07) (Table 5 and Figure 2).

**Table 5:** Meta‐analyses of effect of HIV testing services (HTS) on condom use/condom‐protected sex, by exposure versus comparator and population, 2010 to 2019

Population of Included Studies	Total (N)^a^	Discrete Effects (N)	Effect Size (RR) and 95% CI	Test for Heterogeneity		
				Q	*p*‐value	I^2^ Value (%)
More versus Less Intensive HTS						
All studies	20,264	36	1.03 (0.99 – 1.07)	90.57	<0.0001	57.7
MSM	1,682	7	1.27 (0.92 – 1.76)	41.3	<0.0001	95.8
FSW	4,891	8	1.02 (0.95 – 1.10)	19.7	0.006	63.24
Post‐HTS versus pre‐HTS						
All studies	14,399	50	1.62 (1.33 – 1.99)	1072.8	<0.0001	99.7
Individuals newly diagnosed HIV negative	5,861	16	1.63 (1.01 ‐ 2.62)	188.5	<0.0001	99.7
Individuals newly diagnosed HIV positive	2,713	16	1.65 (1.36 – 1.99)	103.2	<0.0001	92.1
Couples	629	5	5.67 (1.63 – 19.73)	115.7	<0.0001	99.5
MSM	4,845	24	1.23 (1.06 – 1.42)	252.3	<0.0001	97.1
MSM newly diagnosed HIV negative	2008	8	1.06 (0.97 – 1.16)	96.1	<0.0001	87.6
MSM newly diagnosed HIV positive	1,074	11	1.57 (1.25 – 1.96)	36.5	<0.0001	86.7

CI, confidence interval; FSW, female sex worker; HTS, HIV testing services; MSM, men who have sex with men; RR, relative risk.

^a^ Number of individuals included in summary estimate.

These findings were largely consistent for outcomes reported among MSM (RR = 1.27; 95% CI = 0.92 to 1.76) and FSW (RR = 1.02; 95% CI = 0.95 to 1.10). The strongest association between receipt of more‐intensive HTS and increased condom use was in a study of Chinese MSM [91]. In that study, Hao and colleagues found that HIV‐negative MSM randomized to receive HIV testing plus enhanced post‐test counselling, plus a video narrated by HIV‐positive Chinese MSM, plus a bracelet to serve as a reminder to engage in safer sex (more‐intensive HTS) was significantly more likely to report no condomless anal sex with non‐primary partners (RR = 3.5; 95% CI = 2.4 to 5.2) and with primary partners (RR = 1.5; 95% CI = 1.0 to 2.3) compared to those receiving standard HTS.

Thirteen studies were not included in the meta‐analysis because the outcomes could not be pooled or because there were not sufficient data to include in the summary estimate. Eight of these studies observed no significant difference in the overall number of condomless sex acts at follow‐up among those receiving more‐ versus less‐intensive HTS [16, 17, 18, 39, 42, 45, 46, 50], though Metsch and colleagues found that participants who received rapid HIV testing plus individual risk‐reduction counselling (i.e. more‐intensive HTS) reported lower rates of condomless sex with non‐primary partners compared to individuals who received rapid HIV testing with information only (IRR = 0.66; 95% CI = 0.55 to 0.79) [39]. In addition, an RCT by Homsy and colleagues among pregnant women [30] found that HIV‐uninfected pregnant or lactating women in Uganda who received individual or couple‐enhanced counselling quarterly for up to two years post‐partum did not report any difference in the frequency of condom use relative to those receiving standard HTS. The remaining studies reported increases in condom use/decreases in condomless sex for those randomized to more‐intensive versus less‐intensive HTS. A study among African‐American women aged 18 to 65 in the United States found those randomized to more‐intensive HTS reported a similar number of condom‐protected vaginal sex acts at follow‐up compared to less‐intensive HTS, but were significantly more likely to report increasing the frequency of condom use for anal sex [29]. In India, Mimiaga and colleagues observed fewer condomless sex acts at follow‐up among MSM engaged in exchange sex after receiving more‐intensive HTS compared to those receiving less‐intensive HTS [40]. In a US study from Mimiaga and colleagues [41], MSM with crystal methamphetamine dependence who received more‐intensive HTS (enhanced frequency of sexual risk‐reduction counselling) reported significantly fewer condomless anal sex acts with partners who were living with HIV or whose HIV status they did not know at 3‐month follow‐up compared to MSM who received less‐intensive HTS. Wechsberg and colleagues [49] found that South African women who received a gender‐focused HIV prevention intervention in addition to HTS reported more condom use with a main partner compared to women who received only HTS. In an US RCT that enrolled individuals in community supervision programmes and their female sex partners, El‐Bassel and colleagues [24] noted a significantly lower number of condomless sex acts with study partners and non‐study partners for individuals randomized to multi‐session risk‐reduction counselling (more‐intensive HTS) versus one‐time counselling.

#### 3.1.2 Number of sex partners

Twelve studies reported on the number of total sex partners at follow‐up [18, 47, 50]; five [24, 29, 39, 43, 44] were included in meta‐analysis. There was an average of 0.28 fewer sex partners reported at follow‐up among those receiving more versus less‐intensive HTS (mean difference = −0.28; 95% CI = −3.66 to 3.10), though the finding was not significant (*p* = 0.87) and there was significant heterogeneity across studies (χ^2^[df = 9] = 17605, *p* < .0001). In stratified analyses, Oldenburg and colleagues observed that both HIV‐negative and HIV‐positive FSW randomized to more‐intensive HTS reported fewer non‐client sex partners in the past 30 days compared to those randomized to less‐intensive HTS (HIV negative: 3.3 vs. 6.3 partners; HIV positive: 2.2 vs. 9.9 partners) [43].

Three studies noted fewer sex partners [42], fewer new anal sex partners [50], or a lower percentage of reporting multiple male sex partners [47] among participants randomized to more‐intensive HTS compared to less‐intensive HTS. Five studies did not observe significant differences in the mean number of condomless sex partners at follow‐up [18, 32, 39], the number of sex partners [23] or percent reporting at least five clients per day [22] for those receiving more‐ versus less‐intensive HTS. However, in a subgroup analysis, Metsch *et al*. [39] found MSM receiving more‐intensive HTS reported fewer condomless sex partners at follow‐up compared to those receiving less‐intensive HTS (IRR = 0.71; 95% CI = 0.61 to 0.83).

#### 3.1.3 HIV incidence

Eleven studies reported on HIV incidence [17, 22, 25, 26, 28, 30, 33, 35, 39, 45, 91]; ten did not identify statistically significant differences in HIV incidence at follow‐up among individuals receiving more‐ versus less‐intensive HTS. The only study to observe a statistically significant difference was a cluster‐RCT among FSW in Tanzania [33]. In that study, FSW in communities randomized to more‐intensive HTS had a significantly lower HIV incidence compared to FSW in communities with standard HTS (5.0% vs. 10.4% respectively).

#### 3.1.4 STI incidence/prevalence

Eight studies reported on STI incidence, either as a composite STI outcome [32, 36], STI‐specific outcome [22, 23, 45, 91] or both (Table 4) [30, 39]. Only one study observed differences in STI incidence. In that cluster‐RCT in China, Dong and colleagues noted a significant reduction in syphilis diagnoses among FSW in communities randomized to a community‐based comprehensive intervention package compared to control communities (Odds Ratio [OR]=0.51, 95% CI = 0.27 to 0.96) [22]. Although no other studies observed differences in STI incidence, in subgroup analyses Metsch and colleagues observed a higher risk of STI among US MSM randomized to HIV testing plus individual risk‐reduction counselling (i.e. more‐intensive HTS) compared to HIV testing with information only (aRR = 1.41; 95% CI = 1.01 to 1.90) [39].

### 3.2 Exposure/comparator categorization 2: received HTS versus did not receive HTS

Six studies examined outcomes among individuals who received HTS compared to those who did not receive HTS: four were cohort studies [53, 54, 55, 56], one was a cluster‐RCT [52] and one was an individual‐RCT [27] (Table 2). Due to the small number of studies and heterogeneity in how outcomes were reported, we did not conduct meta‐analyses for any outcomes. No study in this category reported on STI incidence.

#### 3.2.1 Condom use

None of the three studies that reported on the condom use observed statistically significant differences between individuals who did and did not receive HTS [27, 52, 54]. Baird and colleagues found that 66% of AGYW who received home‐based HTS reported always using condoms or not having sex at follow‐up compared to 67% among those who did not receive any HTS [52]. In a series of serosurveys, Cawley and colleagues found no differences in the proportion of men and women in Tanzania who reported using condoms with their spouse, non‐primary partner or regular co‐habiting partner among those who did or did not receive HTS [54]. In a cohort study of cross‐border truck drivers in China, Lau *et al*. found no significant differences in condom use with FSWs or non‐primary partners at follow‐up among individuals who did and did not receive HTS [27].

#### 3.2.2 Number of sex partners

Two studies reported on the number of sex partners [52, 54]. In Malawi, a cluster‐RCT of immediate versus delayed home‐based HTS among AGYW (aged 13 to 22) found those receiving immediate home‐based HTS reported a higher mean number of sex partners at follow‐up, though the result was only statistically significant for HIV‐negative participants [52]. In contrast, in Tanzania, HIV‐negative individuals who received HTS were significantly more likely to *decrease* their number of sex partners in the last year between serosurveys, compared to those who did not receive HTS [54].

#### 3.2.3 HIV incidence

Four cohort studies reported on HIV incidence with mixed results [53, 54, 55, 56]. The two studies [53, 55] that compared the frequency of HTS compared to no HTS found individuals undergoing more frequent HIV testing had a higher HIV incidence. Braunstein *et al*. observed that FSW in Rwanda who received HTS at least twice in their lifetime (adjusted Hazard Ratio [aHR]=8.0; 95% CI = 0.9 to 71.3) or once in their lifetime (aHR = 4.2; 95% CI = 0.5 to 39.3) had a higher risk of HIV acquisition than those who had never tested for HIV [53]. Similarly, MSM in the UK who tested for HIV at least quarterly in the past year had a 2.5‐fold higher risk of HIV acquisition in the subsequent year relative to those who had not tested in the past year (aHR = 2.5; 95% CI = 1.3 to 4.7) [55]. Two other cohort studies did not observe a significant difference in HIV incidence by receipt of HTS [54, 56]. However, in a secondary analysis weighted for risk factors, youth who received HTS in South Africa had a significantly lower risk of HIV compared to those never tested (aHR = 0.59; 95% CI = 0.45 to 0.78) [56].

### 3.3 Exposure/comparator categorization 3: post‐HTS versus pre‐HTS

Of 34 studies that examined outcomes among individuals in a period after receiving HTS compared to a period prior to HTS, 32 were cohort studies, one was a study of serial cross‐sectional surveys and one was a two group pre/post‐test quasi‐experimental study (Table 2). None of these studies reported on HIV incidence.

#### 3.3.1 Condom use

**Figure 3:**
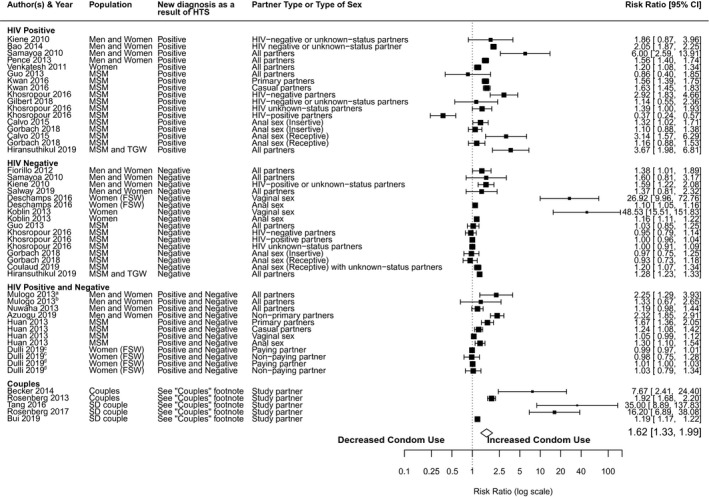
Forest plot of frequency of condom use/condom‐protected sex for studies of post‐HIV testing services (HTS) versus pre‐HTS. CI, confidence interval; FSW, female sex workers; MSM, men who have sex with men; PWID, people who inject drugs; SD, serodiscordant; TGW, Transgender Women. The black squares represent study estimates, the lines 95% CI. The size of the squares represents a study’s weight in the meta‐analysis. The summary effect estimate is displayed as the diamond symbol. Random‐effect model was used to aggregate effect sizes. ^a^Facility‐based HIV testing. ^b^Home‐based HIV testing. ^c^Enhanced HTS. ^d^Standard HTS. Couples: Becker 2014: enrolled couples; partners tested either newly HIV‐positive or newly HIV‐negative (data not dis‐aggregated by HIV test result); Rosenberg 2013: enrolled couples with one partner newly HIV diagnosed; reported condom use with HIV‐negative study partner; Tang 2016: enrolled serodiscordant couples with HIV‐negative spouse newly diagnosed HIV‐negative; Rosenberg 2017: enrolled couples where female partner previously HIV diagnosed, and male partner was newly diagnosed HIV‐positive or ‐negative; Bui 2019 enrolled serodiscordant couples in which one partner tested newly HIV‐positive and one tested newly HIV‐negative.

Thirty‐two studies reported on this outcome [57, 58, 59, 60, 61, 62, 63, 64, 65, 66, 67, 68, 69, 70, 71, 72, 73, 74, 75, 76, 78, 79, 80, 81, 82, 83, 84, 85, 86, 87, 89, 90]. Fifty effect estimates from 27 studies were included in the meta‐analysis (Figure 3). Condom use was significantly higher after receiving HTS compared to before (RR = 1.62; 95% CI = 1.33 to 1.99), with significant heterogeneity across studies (Table 5, Figure 3). The two largest effects in non‐couples’ studies were from studies that enrolled FSW and women at high risk for HIV [65, 76]. Those studies observed substantial increases in condom‐protected vaginal sex (but not anal sex) post‐HTS. The only study to observe an overall significant *decline* in condom use after HIV diagnosis was a US study among MSM [74]. However, the decrease in condom use was only observed among the subset of HIV‐positive MSM with HIV‐positive partners.

When stratifying by HTS outcome, we found that among individuals newly diagnosed HIV positive, condom use was significantly higher after receiving HTS (i.e. after HIV diagnosis) compared to before receiving HTS (RR = 1.65; 95% CI = 1.36 to 1.99) (Table 5). This finding was consistent when we limited the analysis to studies that only included MSM. We observed a marginally significant increase in condom use after HTS among individuals receiving an HIV‐negative diagnosis (RR = 1.63; 95% CI = 1.01 to 2.62). Among the five studies that included serodiscordant couples, condom use was nearly sixfold higher post‐HTS compared to pre‐HTS (RR = 5.67; 95% CI = 1.63 to 19.73).

Five studies were not included in the meta‐analysis [64, 67, 78, 79, 90] because the outcomes were reported in a manner inconsistent with other studies. In Zimbabwe, Cremin and colleagues reported an increase in consistent condom use among individuals testing newly HIV positive compared to before, but the difference was not significant [64]. In Malawi, 28% of individuals newly diagnosed HIV positive reported increasing their condom use since learning their HIV status, but the authors did not report on the statistical significance of this finding [67]. Lin *et al*., noted a substantial increase in condom use among individuals newly diagnosed HIV positive from the time period pre‐HTS (9%) to post‐HTS (91%) [78]. Among individuals newly diagnosed HIV negative, Möller and colleagues found that the odds of condomless anal sex were higher pre‐HTS compared to post‐HTS (aOR = 2.1; 1.2 to 3.6) [79]. Cremin and colleagues did not observe differences in consistent condom use pre‐HTS compared to post‐HTS among HIV‐negative individuals [64]. Finally, Wall and colleagues found that serodiscordant couples in Zambia undergoing couples HTS had significantly fewer condomless sex acts prior to HTS compared to three and six months after HTS [90].

#### 3.3.2 Number of sex partners

The results from the 14 studies that examined this outcome were not summarized in meta‐analyses because of heterogeneity in how outcomes were reported. Nine studies included individuals testing newly HIV positive [58, 60, 64, 70, 72, 77, 86, 88, 89]), seven studies included individuals testing HIV negative [64, 65, 70, 72, 76, 79, 86] and two studies included individuals testing newly HIV positive and negative but did not disaggregate the outcomes by testing status [57, 80].

All nine studies among individuals testing newly HIV positive found that individuals reported fewer sex partners after an HIV‐positive diagnosis versus before, although not all studies identified significant differences. Four studies that included general populations of men and women [58, 64, 86, 89] observed significant declines in the mean number of sex partners after an HIV‐positive diagnosis versus before. However, Venkatesh and colleagues found no difference in the proportion of women reporting >1 sex partner in the past three months before HTS versus after an HIV‐positive diagnosis [89]. Among the four studies that included MSM – two from the USA [70, 88], one from Hong Kong (China) [77] and one from Thailand [72] – all found that the number of sex partners was higher in the period prior to an HIV‐positive diagnosis compared to after. The only study including FSW [60] noted a higher median number of clients before an HIV‐positive diagnosis (median = 10; interquartile range [IQR] = 5 to 18) than after (median = 3; IQR = 2 to 5).

Of the seven studies that compared the number of sex partners reported by individuals before and after testing HIV negative, four included general populations of men and women [64, 65, 76, 86] and three included MSM [70, 72, 79], – all but two [72, 86] observed significant declines in the number of sex partners after an HIV‐negative diagnosis.

The two studies that aggregated outcomes of individuals testing newly HIV positive and newly HIV negative observed mixed results. In a Ugandan study, Mulogo and colleagues noted a reduction in the number of sex partners post‐HTS compared to pre‐HTS [80]; this reduction was larger for those receiving facility‐based versus home‐based HTS. In Nigeria, a study of cantonment residents did not observe a difference in the number of casual sex partners reported at 3‐month post‐HTS compared to the period of time before HTS [57].

#### 3.3.3 STI incidence/prevalence

Two studies reported on STI incidence [62, 72]. Calvo and colleagues noted a higher proportion of MSM and transgender women in Peru was diagnosed with an STI after an HIV‐positive diagnosis (68%) compared to three months before the HIV‐positive diagnosis (47%), though this difference was not statistically significant [62]. In another study of MSM and transgender women in Thailand, Hiransuthikul and colleagues found that the prevalence of any STI declined significantly at 12‐month post‐HTS compared to the time period prior to HTS but only for those individuals who were not diagnosed with HIV. There was no significant change in STI prevalence post‐HTS compared to pre‐HTS among individuals diagnosed with HIV [72].

### 3.4 Risk of bias and quality assessment

Risk of bias assessment for 23 individual‐RCTs revealed that there was low risk of biased allocation to interventions due to inadequate generation of a randomized sequence in all but four studies [27, 30, 42, 50] (Table 6). One of these four studies was a conference abstract [30]. Considering the nature of the intervention that involved interaction between HTS counsellors and individuals who tested for HIV, we assessed a high risk of bias due to blinding of participants and personnel and outcome assessors for the majority of the included individual RCTs. There was a high risk of bias due to incomplete outcome data for eight studies [16, 19, 23, 25, 30, 37, 48, 91], mainly due to a high proportion of missing outcome data that were not balanced across HIV testing and comparator groups.

**Table 6:** Risk of bias assessment of individual randomized controlled trials included in systematic review and meta‐analysis of sexual behaviour change following HTS, 2010 to 2019 (N = 23)

Study	Random Sequence Generation	Allocation concealed	Blinding participants/ personnel	Blinding outcome assessment	Incomplete outcome data	Selective reporting	Other bias
Arnold *et al*., 2019 [16]	Low risk	Low risk	Low risk	Low risk	High risk	Low risk	Low risk
Coffin *et al*., 2014 [18]	Low risk	Low risk	Unclear	Low risk	Low risk	Low risk	Low risk
Crosby *et al*., 2019 [19]	Low risk	Low risk	Low risk	Low risk	High risk	Unclear	Low risk
Duflo *et al*., 2019 [23]	Low risk	Unclear	Low risk	Low risk	High risk	Low risk	Unclear risk
Go *et al*., 2013 [25]	Low risk	Unclear	High risk	Unclear	High risk	Low risk	Low risk
Go *et al*., 2015 [26]	Low risk	Low risk	High risk	Low risk	Low risk	Low risk	Unclear
Hao *et al*., 2012 [91]	Low risk	Low risk	High risk	Low risk	High risk	Low risk	Low risk
Homsy *et al*., 2019 [30]	Unclear	Low risk	High risk	Low risk	High risk	Unclear	Unclear
Jamil *et al*., 2017 [31]	Low risk	Low risk	High risk	Low risk	Low risk	Low risk	Low risk
Katz *et al*., 2018 [32]	Low risk	Low risk	High risk	Low risk	Low risk	Low risk	Low risk
Lau *et al*., 2010 [27]	Unclear	Low risk	High risk	Low risk	Low risk	Low risk	Low risk
Maman *et al*., 2014 [36]	Low risk	Low risk	High risk	Low risk	Low risk	Low risk	Low risk
McMahon *et al*., 2015 [37]	Low risk	Low risk	High risk	Low risk	High risk	Unclear	Low risk
Metsch *et al*., 2012 [38]	Low risk	Low risk	High risk	Low risk	Low risk	Low risk	Low risk
Metsch *et al*., 2013 [39]	Low risk	Low risk	High risk	Low risk	Low risk	Low risk	Low risk
Mimiaga *et al*., 2017 [40]	Low risk	Low risk	High risk	Low risk	Low risk	High risk	Low risk
Mimiaga *et al*., 2019a [41]	Unclear	Unclear	Low risk	Low risk	Low risk	Low risk	High risk
Mimiaga *et al*., 2019b [42]	Low risk	Unclear	Unclear	Low risk	Low risk	Low risk	High risk
Passaro *et al*., 2020 [45]	Low risk	Low risk	Low risk	Low risk	Low risk	Low risk	High risk
Wang *et al*., 2019 [47]	Low risk	Low risk	Low risk	Low risk	Low risk	Low risk	Low risk
Wanyenze *et al*., 2013 [48]	Low risk	Low risk	High risk	Low risk	High risk	Unclear	Low risk
Wray *et al*., 2019 [50]	Unclear	Unclear	High risk	Low risk	Low risk	Low risk	High risk
Zhu *et al*., 2019 [51]	Low risk	Low risk	Low risk	Low risk	Low risk	Low risk	High risk

Risk of bias assessment of 15 cluster‐RCTs revealed low risk of bias for most of the studies except Hawk et. al [29], for which there was evidence of high or unclear risk of bias related to four out of five domains (Table 7). Overall, all the pre‐post studies and cohort studies were judged to be of “good” or “fair” quality, and none were deemed to be of “poor” quality (Tables 8 and 9).

**Table 7:** Risk of bias assessment of cluster randomized controlled trials (RCT) included in systematic review and meta‐analysis of sexual behaviour change following HTS, 2010 to 2019 (N = 15)

Study	Recruitment bias	Baseline imbalance	Loss of clusters	Incorrect analysis	Comparability with Individual RCTs
Baird *et al*., 2014 [52]	Low risk	Low risk	Low risk	Low risk	Low risk
Coates *et al*., 2014 [17]	Low risk	Low risk	Low risk	Low risk	Low risk
Daniels *et al*., 2014 [20]	Low risk	Low risk	Low risk	Low risk	Low risk
Doherty *et al*., 2013 [21]	Low risk	Unclear	Low risk	Low risk	Low risk
Dong *et al*., 2019 [22]	Low risk	Unclear	High risk	Low risk	Low risk
El‐Bassel *et al*., 2019 [24]	Low risk	Low risk	High risk	Low risk	Unclear
Havlir *et al*., 2019 [28]	Low risk	Low risk	High risk	Low risk	Low risk
Hawk *et al*., 2013 [29]	High risk	Low risk	High risk	High risk	Unclear
Kerrigan *et al*., 2019 [33]	Low risk	Unclear	Low risk	Low risk	Low risk
Kuteesa *et al*., 2019 [34]	Low risk	Low risk	Low risk	High risk	High risk
Makhema *et al*., 2019 [35]	Low risk	Low risk	Low risk	Low risk	Low risk
Oldenburg *et al*., 2018 [43]	Low risk	Low risk	Low risk	Low risk	Unclear
Ortblad *et al*., 2019 [44]	Low risk	Low risk	Low risk	Low risk	Unclear
Starks *et al*., 2019 [46]	Low risk	Low risk	Low risk	Low risk	Unclear
Wechsberg *et al*., 2019 [49]	Low risk	High risk	Low risk	Low risk	Low risk

**Table 8:** Quality assessment of studies reporting on outcomes after (post) versus before (pre) receiving HIV Testing Services (HTS) included in systematic review and meta‐analysis of sexual behaviour change following HTS, 2010 to 2019 (N = 34)

Study	C1	C2	C3	C4	C5	C6	C7	C8	C9	C10	C11	C12	Overall quality rating
Azuogu *et al*., 2019 [57]	Yes	No	Yes	CD	Yes	Yes	Yes	No	Yes	No	No	No	Fair
Bao *et al*., 2014 [58]	Yes	Yes	Yes	Yes	Yes	Yes	Yes	No	Yes	No	No	NA	Fair
Becker *et al*., 2014 [59]	Yes	Yes	Yes	No	Yes	Yes	Yes	No	No	Yes	No	NA	Fair
Braunstein *et al*., 2011 [60]	Yes	Yes	Yes	No	Yes	Yes	Yes	No	Yes	No	No	NA	Fair
Bui *et al*., 2019 [61]	Yes	Yes	Yes	Yes	Yes	Yes	Yes	No	Yes	No	No	No	Fair
Calvo *et al*., 2015 [62]	Yes	No	Yes	CD	No	Yes	Yes	CD	Yes	Yes	No	NA	Fair
Coulaud *et al*., 2019 [63]	Yes	No	Yes	CD	Yes	Yes	Yes	No	No	No	No	NA	Fair
Cremin *et al*., 2010 [64]	Yes	Yes	Yes	No	Yes	Yes	Yes	No	CD	Yes	Yes	Yes	Good
Deschamps *et al*., 2016 [65]	Yes	Yes	Yes	CD	Yes	Yes	Yes	No	Yes	Yes	No	NA	Fair
Dulli *et al*., 2019^a^ [66]	Yes	Yes	Yes	CD	Yes	Yes	Yes	No	Yes	Yes	No	No	Fair
Fedor *et al*., 2015 [67]	Yes	Yes	Yes	CD	Yes	Yes	No	CD	Yes	Yes	No	NA	Fair
Fiorillo *et al*., 2012 [68]	Yes	Yes	Yes	No	Yes	Yes	No	No	Yes	Yes	No	NA	Fair
Gilbert *et al*., 2018 [69]	Yes	Yes	Yes	No	No	Yes	Yes	No	Yes	No	Yes	NA	Fair
Gorbach *et al*., 2018 [70]	No	Yes	Yes	CD	No	Yes	Yes	No	No	Yes	No	NA	Fair
Guo *et al*., 2013 [71]	Yes	Yes	No	Yes	No	No	Yes	No	Yes	No	No	NA	Fair
Hiransuthikul *et al*., 2019 [72]	Yes	Yes	Yes	CD	Yes	Yes	Yes	No	No	Yes	No	NA	Fair
Huan *et al*., 2013 [73]	Yes	No	Yes	No	Yes	Yes	Yes	No	No	Yes	No	NA	Fair
Khosropour *et al*., 2016 [74]	Yes	Yes	Yes	Yes	No	Yes	Yes	No	No	Yes	No	NA	Fair
Kiene *et al*., 2010 [75]	Yes	Yes	Yes	No	Yes	Yes	Yes	No	Yes	Yes	No	NA	Fair
Koblin *et al*., 2013 [76]	Yes	Yes	Yes	Yes	Yes	Yes	Yes	Yes	No	Yes	No	NA	Good
Kwan *et al*., 2016 [77]	Yes	Yes	Yes	No	Yes	Yes	Yes	No	Yes	No	No	No	Fair
Lin *et al*., 2013 [78]	Yes	Yes	Yes	No	CD	Yes	Yes	No	No	No	No	NA	Fair
Möller *et al*., 2015 [79]	Yes	Yes	Yes	Yes	Yes	Yes	Yes	No	No	Yes	No	NA	Fair
Mulogo *et al*., 2013 [80]	Yes	No	Yes	No	Yes	Yes	Yes	No	No	Yes	No	NA	Fair
Nuwaha *et al*., 2013^a^ [81]	Yes	Yes	Yes	No	Yes	No	Yes	No	NA	NA	No	Yes	Fair
Pence *et al*., 2013 [82]	Yes	No	Yes	CD	Yes	Yes	Yes	No	No	No	No	NA	Fair
Rosenberg *et al*., 2013 [83]	Yes	Yes	Yes	Yes	Yes	Yes	Yes	No	Yes	No	No	NA	Fair
Rosenberg *et al*., 2017 [84]	Yes	Yes	Yes	No	Yes	Yes	No	No	Yes	Yes	No	NA	Fair
Salway *et al*., 2019 [85]	Yes	No	Yes	No	No	Yes	Yes	CD	Yes	Yes	No	No	Fair
Samayoa *et al*., 2010 [86]	No	No	Yes	Yes	No	Yes	Yes	No	No	Yes	No	NA	Fair
Tang *et al*., 2016 [87]	Yes	No	Yes	No	CD	Yes	Yes	No	Yes	Yes	No	NA	Fair
Vallabhaneni *et al*., 2013 [88]	Yes	Yes	Yes	No	No	Yes	Yes	No	No	Yes	No	NA	Fair
Venkatesh *et al*., 2011 [89]	Yes	Yes	No	No	Yes	Yes	Yes	Yes	Yes	Yes	No	NA	Fair
Wall *et al*., 2016 [90]	Yes	No	CD	CD	Yes	Yes	Yes	No	CD	Yes	No	NA	Fair

C1. Was the study question or objective clearly stated?. C2. Were eligibility/selection criteria for the study population prespecified and clearly described? C3. Were the participants in the study representative of those who would be eligible for the test/service/intervention in the general or clinical population of interest? C4. Were all eligible participants that met the prespecified entry criteria enrolled? C5. Was the sample size sufficiently large to provide confidence in the findings? C6. Was the test/service/intervention clearly described and delivered consistently across the study population? C7. Were the outcome measures prespecified, clearly defined, valid, reliable and assessed consistently across all study participants? C8. Were the people assessing the outcomes blinded to the participants' exposures/interventions? C9. Was the loss to follow‐up after baseline 20% or less? Were those lost to follow‐up accounted for in the analysis? C10. Did the statistical methods examine changes in outcome measures from before to after the intervention? Were statistical tests done that provided *p* values for the pre‐to‐post changes? C11. Were outcome measures of interest taken multiple times before the intervention and multiple times after the intervention (i.e. did they use an interrupted time‐series design)? C12. If the intervention was conducted at a group level (e.g. a whole hospital, a community, etc.) did the statistical analysis take into account the use of individual‐level data to determine effects at the group level? CD, cannot determine; NA, not applicable; NR, not reported.

^a^ All studies included in pre/post analysis are cohort studies by design except Nuwaha 2013 (serial cross‐sectional) and Dulli 2019 (Two‐group, pre‐/post‐test quasi experiment).

**Table 9:** Risk of bias assessment for cohort studies included in received HIV testing services (HTS) versus did not receive HTS for systematic review and meta‐analysis of sexual behaviour change following HTS, 2010 to 2019 (N = 4)

Study	C1	C2	C3	C4	C5	C6	C7	C8	C9	C10	C11	C12	C13	C14	Overall quality rating
Braunstein *et al*., 2011 [53]	Yes	Yes	Yes	Yes	No	Yes	Yes	Yes	Yes	No	Yes	No	Yes	Yes	Good
Cawley *et al*., 2014 [54]	Yes	Yes	Yes	No	No	Yes	Yes	No	No	No	Yes	No	No	Yes	Fair
Furegato *et al*., 2018 [55]	No	Yes	Yes	Yes	No	Yes	Yes	No	Yes	Yes	Yes	No	Yes	Yes	Fair
Rosenberg *et al*., 2013 [56]	Yes	Yes	CD	Yes	No	No	Yes	Yes	Yes	Yes	Yes	No	Yes	Yes	Fair

C1. Was the research question or objective in this paper clearly stated? C2. Was the study population clearly specified and defined? C3. Was the participation rate of eligible persons at least 50%? C4. Were all the subjects selected or recruited from the same or similar populations (including the same time period)? Were inclusion and exclusion criteria for being in the study prespecified and applied uniformly to all participants? C5. Was a sample size justification, power description, or variance and effect estimates provided? C6. For the analyses in this paper, where the exposure(s) of interest measured prior to the outcome(s) being measured? C7. Was the timeframe sufficient so that one could reasonably expect to see an association between exposure and outcome if it existed? C8. For exposures that can vary in amount or level, did the study examine different levels of the exposure as related to the outcome (e.g. categories of exposure, or exposure measured as a continuous variable)? C9. Were the exposure measures (independent variables) clearly defined, valid, reliable and implemented consistently across all study participants? C10. Was the exposure(s) assessed more than once over time? C11. Were the outcome measures (dependent variables) clearly defined, valid, reliable and implemented consistently across all study participants? C12. Were the outcome assessors blinded to the exposure status of participants? C13. Was loss to follow‐up after baseline 20% or less? C14. Were key potential confounding variables measured and adjusted statistically for their impact on the relationship between exposure(s) and outcome(s). CD, cannot determine; NA, not applicable; NR, not reported.

## 4 DISCUSSION

In this review of sexual behaviour change following HTS, we found that receipt of more‐intensive HTS was not significantly associated with subsequent increases in condom use relative to less‐intensive HTS. Likewise, we did not observe differences in subsequent condom use among individuals who did and did not receive HTS. However, we found that receipt of HTS was significantly associated with increases in condom use after receipt of HTS among individuals newly diagnosed HIV positive, but only marginally significant among individuals diagnosed HIV negative. This finding was consistent when we stratified studies to include only MSM or only serodiscordant couples. Taken together, these results suggest that enhanced counselling or other components included in more‐intensive HTS may not have a large impact on subsequent sexual behaviour, but that receipt of HTS may affect subsequent sexual behaviour among individuals diagnosed HIV positive.

The finding that more‐intensive HTS was not associated with changes in behaviour or HIV/STI incidence is noteworthy. For the majority of these studies, the “more” intensive intervention included additional or enhanced counselling sessions or support groups above and beyond that which is included within pre‐test information in standard HTS, and thus our findings indicate that these additional counselling components may not have a strong influence on subsequent sexual behaviour or HIV/STI incidence. Furthermore, some of the more‐intensive HTS may be difficult or expensive to implement at a population‐level (e.g. HTS with multi‐session behavioural counselling); thus, in a setting of limited resources, these more‐intensive HTS interventions may not provide large gains in HIV prevention for the cost. That said, there were several studies that observed lower rates of condomless sex, increases in condom use, or fewer sex partners among key populations [29, 39, 40, 43, 91], indicating that more‐intensive HTS may be beneficial for some populations or within some settings.

We included HIV and STI incidence as an outcome in this review as a proxy for sexual behaviour change. Given that we did not observe significant differences in condom use comparing more‐intensive HTS versus less‐intensive HTS, it is not surprising that most studies did not observe significant differences in HIV or STI incidence among those two groups. However, three community‐based HTS studies demonstrated a slight reduction in HIV incidence [17, 21] or a significant reduction in HIV incidence [33] among communities randomized to more‐intensive HTS. But these studies involved multi‐component interventions; thus, it is difficult to disentangle the impact of an individual component. Of the studies that compared the receipt of any HTS to no HTS, two [53, 55] examined the frequency of HTS and found those testing more frequently had a higher subsequent risk of HIV acquisition. However, it remains unclear if this is due to more frequent testing (i.e. “the more you test, the more you find”), due to the fact that those individuals at high risk for HIV tested more frequently, or that there is minimal or no impact of HTS on subsequent behaviour change and corresponding risk of HIV acquisition.

A key finding of this review is that individuals substantially change their condom use behaviour after being diagnosed with HIV, a finding that confirms the previous meta‐analysis by Fonner and colleagues [10]. Our results were consistent when we calculated stratified estimates for MSM and couples. In contrast, we only observed a marginally statistically significant change in condom use after an HIV‐negative diagnosis. Given that our review is the third to demonstrate that receiving an HIV‐negative diagnosis does not lead to substantial increase in condom‐protected sex, our results suggest that it is the HIV‐positive diagnosis, not the testing itself, that likely has an impact on subsequent behaviour, and somewhat calls into question the notion that HIV testing itself (in the absence of an HIV diagnosis) directly leads to modifications in sexual behaviour. This finding also highlights the importance of integrating PrEP referrals or PrEP provision within HTS as part of an HIV prevention package for individuals diagnosed HIV negative who may be at ongoing HIV risk.

From a public health standpoint, the modification in behaviour immediately following a new HIV diagnosis is critically important, as it has the potential to reduce HIV transmission to HIV‐uninfected partners at a time when individuals newly diagnosed with HIV may not yet be linked to ongoing HIV medical care or may not yet be virally suppressed. However, in our meta‐analysis we only used outcome data from studies’ first follow‐up time point, which was typically between three and twelve months after the HIV‐diagnosis. So, although we observed an immediate change in behaviour following an HIV‐positive diagnosis, it remains unclear if the change is durable beyond a few months. Several studies included in this review have observed subsequent decreases in condom use after the initial increases observed immediately following HIV diagnosis [69, 74, 77, 82]. In the era of undetectable–untransmissible, future studies will also likely start to observe longer term decreases in condom use among these populations. Our findings therefore confirm the importance of immediate linkage to ART and sustained engagement in HIV care to achieve continuous viral suppression.

The largest effect of sexual behaviour change following HIV diagnoses that we observed was among couples, where there was a nearly sixfold increase in condom use after HTS. These studies varied in the type of HTS offered (i.e. couples HTS [59, 84] vs. standard HTS [83, 87]), and in the HIV status of enrolled couples. Despite these differences, the increase in condom use was large and relatively consistent across the studies, suggesting that the effect of HTS on subsequent sexual behaviour is particularly robust among couples, and underscores the importance of couples HTS programmes.

There are several notable limitations. First, there was marked variation in study outcome measures, particularly in terms of the follow‐up time‐points and recall periods, which prevented an assessment of behavioural outcomes for different time intervals. Second, the outcome of condom‐protected sex was not clearly labelled in some studies and was obtained by inverting the outcome of “condomless sex.” Doing so might not have always precluded “abstinence” from condom‐protected sex and hence, the outcome might not have always been consistently extracted across studies. Third, there was a high degree of heterogeneity, supported by the heterogeneity test in our meta‐analyses (Table 5). While we acknowledge this challenge, we were unable to explore the source of additional heterogeneity beyond the a *priori* subgroup and stratified analyses. Fourth, the majority of pre‐post studies did not always include the same number of people in the post‐ and the pre‐HTS group. Fifth, we did not consider the adjusted estimates that were reported, and instead utilized raw Ns from the studies to calculate summary estimates. Sixth, the decision to meta‐analyse estimates from observational studies may be prone to bias including confounding; however, we carefully considered the quality and methodological homogeneity of these studies prior to meta‐analysis. Sixth, we cannot exclude the possibility of recall bias or social‐desirability bias for self‐reported outcomes. Seventh, we did not extract data such as individuals’ or partners’ PrEP use, ART use or viral load, or changes in seroadaptive behaviours, all of which could affect an individual’s decision about whether or not to use condoms. Eight, this review only included testing services related to HIV, and not STI, though some of the “more‐intensive” HTS may have included STI testing. Ninth, there is heterogeneity in the goals of HTS, and it is possible that not all HTS explicitly have a goal of modifying subsequent condom use and number of sex partners. Finally, we did not assess publication bias as part of this review but note that this review only included studies published in peer‐reviewed literature or accepted as conference abstracts.

## 5 CONCLUSIONS

Our review indicates that enhanced counselling or other components often included in more‐intensive HTS may not have a large impact on condom use, but that receipt of an HIV‐positive diagnosis likely affects condom use, at least in the time period immediately following an HIV diagnosis. These results provide reassurance that most populations increase condom use during a period in which they may not yet be fully engaged in HIV care or virally suppressed. Among most populations, we did not observe changes in sexual behaviour after being diagnosed HIV negative. This underscores the need to incorporate PrEP provision or PrEP referral services into HTS. The findings from this review suggest that limited HTS resources should be focused on expanding efforts that promote early HIV diagnosis and linkage to treatment and prevention services instead of more‐intensive approaches that incorporate enhanced behavioural counselling.

## COMPETING INTERESTS

CMK has received donations of specimen collection kits and reagents from Hologic, Inc. for studies unrelated to the submitted work.

## AUTHORS’ CONTRIBUTIONS

CMK and BB developed the initial search strategy with input from CJ, MSJ, RB, MBD, KS. CMK, RT, JW, HH, BB, DK, NK, ATT, KAC, SF, BG and MA were involved in title and abstract screening. RT, NK and LS extracted the data. CMK verified eligibility of the included studies, verified extracted data and resolved discrepancies at each step of the review. RT conducted the analysis with input from CMK. RT and CMK wrote the initial draft of the manuscript. All authors critically reviewed and commented on the drafts and approved the final version of the manuscript.

## Supporting information


**Data S1.** Full search strategy.
