# Supplementary material for: Sexual behaviour change following HIV testing services: a systematic review and meta‐analysis
Source: J Int AIDS Soc. 2020 Nov 8;23(11):e25635. doi: 10.1002/jia2.25635 (PMC7649006; doi:10.1002/jia2.25635)
Supplement: Supplementary file 1 — Data S1. Full search strategy. [file JIA2-23-e25635-s001.docx]

**Supplement 1.** Full search strategy

Note:

1. Database searched: PubMed/Medline, PsycINFO, Cochrane Library, CINAHL, Web of Science, EMBASE, Global Health Database, ICTRP, and PACTR
2. To identify conference abstracts not indexed in the electronic databases, we searched International AIDS Society 2017 and 2018 and Conference on Retroviruses and Opportunistic Infections 2018 and 2019 abstract electronic databases and/or abstract books.
3. Only terms for “counselling and testing” were used to search conference abstracts because all conference being searched were HIV-related and search functions were limited.
4. The search strategy below is in PubMed format

**Component 1: Counselling/Behaviour Change**

(

(“HIV Incidence”[tiab] OR “human immunodeficiency virus incidence”[tiab] OR “incidence of human immunodeficiency virus”[tiab] OR “incidence of HIV”[tiab] )

OR

(“sexually transmitted infection incidence”[tiab] OR “incidence of sexually transmitted disease”[tiab] OR “incidence of sexually transmitted infection”[tiab] OR “sexually transmitted disease incidence”[tiab] OR (“Sexually Transmitted Diseases”[MeSH] AND “Incidence”[MeSH]) OR "sti incidence"[tiab] OR "std incidence"[tiab])

OR

((“Pregnancy”[MeSH] OR “pregnancy”[tiab]) AND (“incidence”[tiab] OR “incidence”[MeSH] OR “cohort”[tiab] OR “prospective”[tiab] OR “follow-up”[tiab] OR “pre- and post”[tiab] OR “longitudinal”[tiab]))

OR

(“positive life events”[tiab] OR “negative life events”[tiab] OR “Life Change Events”[MeSH] OR “Family Conflict”[MeSH] OR “Interpersonal Relations”[MeSH] OR “Stress, Psychological”[MeSH] OR “Spouse Abuse”[MeSH] OR “Sex Offenses”[MeSH] OR “Violence”[MeSH] OR "Substance-Related Disorders"[Mesh] OR “sexual relationship”[tiab] OR "sexual relationships"[tiab] OR “physical abuse”[tiab] OR “emotional abuse”[tiab])

OR

(“psychosocial outcomes”[tiab] OR “Personal Autonomy”[MeSH] OR “Social Stigma”[MeSH] OR “Social Environment”[MeSH] OR “self-efficacy”[tiab] OR “stigma”[tiab] OR “social support”[tiab])

OR

(“Contraceptive Devices, Male”[MeSH] OR “Condoms, Female”[MeSH] OR “condom-protected sex”[tiab] OR “unprotected intercourse”[tiab] OR “unprotected sex”[tiab])

OR

(“Coitus”[MeSH] OR “Extramarital Relations”[MeSH] OR “Safe Sex”[MeSH] OR “Unsafe Sex”[MeSH] OR “safe sex”[tiab] OR “unsafe sex”[tiab] OR “Sexual Partners”[MeSH] OR “high-risk behaviour”[tiab] OR “extramarital sexual activity”[tiab] OR “extramarital sex”[tiab] OR “Risk Reduction Behaviour”[MeSH] OR “number of new partners”[tiab] OR “Risk-Taking”[MeSH] OR “number of sexual partners”[tiab] OR “Sex Work”[MeSH])

OR

(“voluntary medical male circumcision” or “circumcision” or “male circumcision” or “VMMC” or “pre-exposure prophylaxis” or “OST” or “opioid substitution therapy”)

)

AND

**Component 2: Study Type**

("Evaluation Studies" [Publication Type] OR "Evaluation Studies as Topic"[Mesh] OR "Clinical Study" [Publication Type] OR “Pilot Projects”[MeSH] OR “Risk Assessment”[MeSH] OR “Longitudinal Studies”[MeSH] OR “Comparative Effectiveness Research”[MeSH] OR “Community-Based Participatory Research”[MeSH] OR “control groups”[MeSH] OR “Matched-Pair Analysis”[MeSH] OR “Clinical Studies as Topic”[MeSH] OR “Epidemiologic Studies”[MeSH] OR “Patient Reported Outcome Measures”[MeSH] OR “Focus Groups”[MeSH] OR “Interviews as topic”[MeSH] OR “Records as topic”[MeSH] OR "Comparative Study" [Publication Type] OR “Program Evaluation”[MeSH] OR “Outcome and Process Assessment (Health Care)”[MeSH] OR “Prevalence”[MeSH] OR “Incidence”[MeSH] OR “cohort”[tiab] OR “prospective”[tiab] OR “follow-up”[tiab] OR “followup”[tiab] OR “incidence”[tiab] OR “prevalence” OR “longitudinal”[tiab] OR “evaluation”[tiab])

AND

**Component 3: HIV Testing**

(

("HIV Infections/diagnosis"[Mesh] OR "AIDS Serodiagnosis"[Mesh])

OR

(("HIV Infections"[Mesh] OR “HIV”[mesh] OR HIV [tiab] OR HIV1[tiab] OR HIV2[tiab] OR “human immunodeficiency virus” [tiab] OR “human immunedeficiency virus” [tiab] OR “human immuno deficiency virus” [tiab] OR “human immune deficiency virus” [tiab] OR ((human immun*[tiab]) AND (deficiency virus[tiab])) OR "Acquired Immunodeficiency Syndrome"[Mesh] OR ((acquired immun*[tiab]) AND (deficiency syndrome[tiab])) OR “acquired immunodeficiency syndrome”[tiab] OR “acquired immunedeficiency syndrome”[tiab] OR “acquired immuno-deficiency syndrome”[tiab] OR “acquired immune-deficiency syndrome”[tiab])

AND

("Diagnostic Tests, Routine"[Mesh] OR "Mass Screening"[Mesh] OR diagnos*[tw] OR serodiagnos*[tw] OR test[tiab] OR tests[tiab] OR testing[tiab] OR screening[tiab]))

)
